# Unveiling the Value of *Amomum tsaoko* Crevost & Lem.: A Review from Bioactive Compounds to Health Benefits and Industrial Applications

**DOI:** 10.3390/foods15142513

**Published:** 2026-07-16

**Authors:** Yaling Pu, Jingjing Wu, Chuandi Liu, Ziqiao Xu, Kun Liu, Haonan Zhang, Yongcheng Yang, Conglong Xia

**Affiliations:** 1 College of Pharmacy, Dali University, Dali 671000, China; pyaling1002@163.com (Y.P.); 19869017294@163.com (J.W.); 15966121609@163.com (C.L.); xzq578440737@163.com (Z.X.); 15908747920@163.com (K.L.); zhn2504612109@gmail.com (H.Z.); 2 Yunnan International Joint Laboratory of Characteristic Medicinal and Edible Resources, Dali 671000, China

**Keywords:** *Amomum tsaoko* Crevost & Lem., functional components, bioactivities, applications, safety assessment

## Abstract

*Amomum tsaoko* Crevost & Lem. (AT) is a representative edible, medicinal spice widely used in Southeast Asia for food seasoning and flavor enhancement. Growing evidence suggests that it is a rich source of bioactive phytochemicals with diverse health-promoting properties, necessitating a systematic synthesis of its functional attributes and underlying mechanisms to better guide future applications. AT has been reported to contain flavonoids, diarylheptanoids, phenolic acids, terpenoids, steroids, and volatile oils. These constituents are associated with a broad spectrum of biological activities, including antimicrobial, antidiabetic, antioxidant, anti-inflammatory, anticancer, neuroprotective, anti-obesity, gastrointestinal protective, and immunomodulatory effects. Due to its functional properties and economic value, AT shows considerable potential for application in functional foods, pharmaceuticals, cosmetics, and agriculture. However, despite its extensive utilization, integrated reviews that systematically link bioactivities, toxicological evidence, and industrial applications remain scarce. This review comprehensively summarizes recent advances in the bioactive compounds, health functions, toxicological evaluation, and industrial applications of AT. Current research progress, key limitations, and future perspectives are critically discussed. Additionally, by providing a comprehensive overview of its multifaceted benefits and applications, this review fills an important gap and offers insights to support further research and multi-sectoral exploitation of AT.

## 1. Introduction

**Figure 1:**
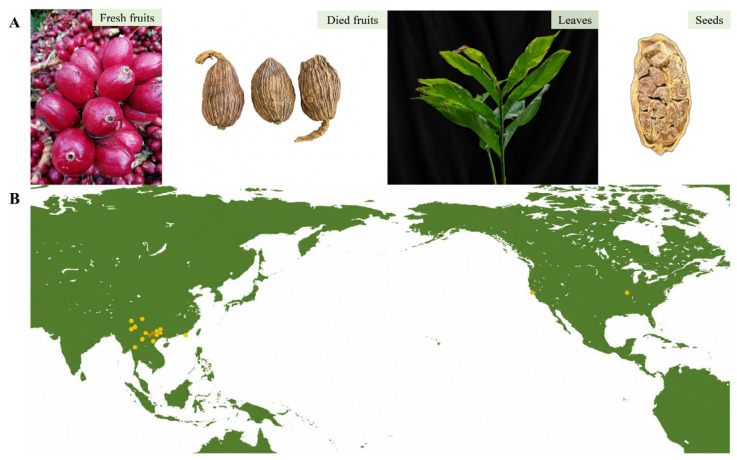
The fresh fruits, dried fruits, leaves and seeds of *Amomum tsaoko* Crevost & Lem. (**A**). The global distribution map of *Amomum tsaoko* Crevost & Lem. (adapted from the Global Biodiversity Information Facility database, https://www.gbif.org/, accessed on 11 June 2026) (**B**).

*Amomum tsaoko* Crevost & Lemarié (AT), a perennial species belonging to the genus Amomum (*Zingiberaceae*), is predominantly distributed across Southwest China, northern Vietnam, and other parts of Asia [1] (Figure 1). China is the primary production region for AT, with Yunnan Province accounting for more than 95% of the national cultivation area [2]. The dried ripe fruits of AT have long been valued for their dual roles as both a culinary spice and a medicinal resource. In Chinese cuisine, the fruits are commonly added to meat dishes, hotpots, and soups to enhance flavor and mask undesirable odors, an effect primarily attributed to their rich volatile constituents [3]. In traditional Chinese medicine, AT has been extensively used to treat disorders associated with cold–dampness affecting the spleen and stomach, as well as infectious diseases such as malaria [4]. Its medicinal use can be traced back to the Song Dynasty, where it was recorded in the classical medical text *Taiping Huimin Heji Jufang* [5]. Furthermore, AT is officially listed in the Pharmacopoeia of the People’s Republic of China 2025, which records various prescriptions containing this species [6].

With advances in natural product chemistry and functional food research, increasing attention has been directed toward the phytochemical composition and biological activities of AT. To date, numerous bioactive constituents have been isolated and characterized from AT, including phenolics, flavonoids, diarylheptanoids, bicyclic nonanes, and steroids. These compounds exert diverse pharmacological and health-promoting effects through multiple molecular targets and complex mechanisms, including the regulation of gastrointestinal function, antimicrobial and anti-inflammatory activities, anticancer effects, and neuroprotection. These characteristics are closely linked to the health benefits of AT [7]. Nevertheless, current research has largely focused on the isolation and bioactivity assessment of constituents derived from the fruits and essential oils, whereas comprehensive investigations of other plant tissues and non-volatile constituents remain scarce. In parallel, growing interest has been directed toward the utilization of AT in the food industry, particularly as a natural preservative, flavoring agent, and functional ingredient. Despite these promising applications, challenges related to stability, standardization, and industrial-scale implementation remain unresolved. Furthermore, the expanding use of AT has highlighted the importance of comprehensive safety evaluation. Although AT has long been regarded as safe in traditional use, there remains a lack of comprehensive evaluation of its safety and toxicological profile.

Therefore, this review aims to provide a systematic summary of the bioactive compounds, health functions, application potential, and safety profile of AT. Based on this summary, we discuss the limitations of current research and outline future directions, with the goal of providing guidance for its further development in food and health-related fields.

## 2. Methodology

This review comprehensively summarizes the current knowledge regarding the phytochemical constituents, pharmacological activities, structure–activity relationships, industrial applications, and safety of AT based on a systematic literature search. Relevant literature was primarily retrieved from the following major international databases: Web of Science, PubMed, ScienceDirect, Google Scholar, Scopus, and CNKI. The search covered publications from database inception to March 2026, with particular emphasis on studies published after 2020. Boolean operators (AND/OR) were combined with targeted keywords, including: “*Amomum tsaoko*”, “Chemical composition”, “essential oil”, “volatile compounds”, “bioactive compounds”, “pharmacological activity”, “aromatic components”, “diarylheptanoids”, “flavonoids”, “structure-function relationship”, “application”, “toxicity”, and “safety”. Priority was given to original research articles published in peer-reviewed journals, while authoritative review articles were consulted to provide background information and identify additional relevant studies. Patent literature was additionally consulted to support the discussion of industrial applications and technological developments. Master’s and doctoral theses were considered for inclusion only when they reported original experimental findings unavailable in the peer-reviewed literature and provided essential evidence relevant to specific topics discussed in this review. Studies lacking sufficient experimental evidence, non-peer-reviewed conference abstracts, and non-English publications with limited accessibility were excluded. The titles and abstracts of the retrieved records were initially screened for relevance. Subsequently, the full texts of potentially eligible publications were assessed according to the predefined inclusion and exclusion criteria, and the selected studies were categorized according to the major topics of this review, including phytochemistry, pharmacological activities, structure–activity relationships, industrial applications, and safety.

## 3. Chemicals

### 3.1. Nutrients

As a popular spice in food processing, AT also possesses certain nutritional value. FAT has been reported to contain eight saturated fatty acids, with palmitic acid (125–181 mg/kg) being the most abundant. Furthermore, five unsaturated fatty acids have also been identified in FAT, with oleic acid (120–181 mg/kg) being the most abundant, followed by linoleic acid (54.3–113 mg/kg) and linolenic acid (18.7–45.3 mg/kg) [8]. Notably, as drying progresses, the content of most fatty acids initially decreases before subsequently increasing, reaching a relatively high concentration after 24 h of oven drying at 60 °C. This trend is likely attributable to the accumulation of fatty acids caused by progressive moisture loss during drying [8]. In addition to lipid components, AT also contains polysaccharides. Crude polysaccharides (ATP) were isolated from AT via hot water extraction and ethanol precipitation, with a yield of 5.22% (dry weight basis). Subsequent purification resulted in the isolation of ATP-4, a novel acidic heteropolysaccharide with immunomodulatory activity. Its monosaccharide composition includes rhamnose (Rha), galacturonic acid (GalA), glucose (Glc), galactose, xylose (Xyl), and arabinose, with a molar ratio of 9.03:52.37:7.89:12.34:6.47:11.89 and a molecular weight of 4.23 × 10^4^ Da. Structural characterization revealed that the backbone of ATP-4 is predominantly composed of 1,4-linked GalpA (47.87%), displaying characteristic homogalacturonan regions interspersed with rhamnogalacturonan domains (1,2-linked-Rhap and 1,2,4-linked-Rhap). The presence of 1,3-linked-Glcp, T-Glcp, and 1,4-linked-Xylp linkages further differentiates ATP-4 from conventional pectic polysaccharide structures. Furthermore, the ATP-4 degradation product AO-2 and the modified polysaccharide ATP-4e have demonstrated enhanced immunomodulatory activity [9].

### 3.2. Bioactive Compounds

Recent advances in analytical methodologies and structural characterization techniques have greatly facilitated the systematic investigation of secondary metabolites in AT, thereby broadening their potential applications. To date, at least 493 structurally diverse compounds have been identified from AT [7], including flavonoids, diphthalides, phenolic acids, terpenes, steroids, and aroma components. Detailed information on these compounds is provided in Table S1.

#### 3.2.1. Aromatic Active Components

AT is characterized by its distinctive aromatic and pungent properties, which are primarily attributed to its volatile constituents (essential oils). A large number of aromatic components include monoterpenes, oxygenated monoterpenes, sesquiterpenes, oxygenated sesquiterpenes, esters, aldehydes, and alcohols. Among these, aldehydes represent the most abundant class of aromatic components (2607 to 3848 mg/kg) and significantly contribute to the overall aromatic profile [8]. Numerous studies have demonstrated that various factors, including storage duration, harvest season, extraction method, geographical distribution, and drying method, influence the composition and abundance of aromatic compounds in AT. For instance, during natural drying, while the number of aromatic compounds remains constant, their concentrations undergo dynamic changes [10]. In contrast, oven drying at 50 °C significantly reduces drying time while preventing mold growth and fruit cracking, effectively retaining high levels of essential oils and volatile compounds in AT [11]. In terms of controllability and overall aroma acceptance, oven drying offers distinct advantages and is therefore considered more suitable for industrial processing [12]. In addition, the length of AT fruits and seeds is positively correlated with both aroma intensity and biological activity [13]. It has been reported that the differences in aroma profiles between FAT and DAT are mainly attributed to variations in the concentrations of aldehydes and terpenes. Specifically, drying leads to a significant reduction in aldehyde levels, while terpene levels increase. Consequently, DAT generally exhibits a stronger fatty and spicy aroma, making it a popular spice [14]. In terms of extraction techniques, Cui et al. employed a green and efficient modified solvent-free microwave extraction (M-SFME) method for the extraction of AEO. Under optimal conditions, the yield reached 1.13%, significantly surpassing that achieved by conventional steam distillation (0.84%) and conventional SFME (0.91%). Furthermore, M-SFME offers advantages such as a short extraction time, low energy consumption, and minimal environmental impact, making it a promising alternative technology for the extraction of essential oils from AT and other aromatic plants [15]. Another study utilized two methods, hydrodistillation (HD) and solvent extraction combined with solvent-assisted flavor evaporation (SE-SAFE), to isolate the volatile components of AT cobs to evaluate the influence of extraction techniques on key odor-active compounds. The results indicated that most aromatic active compounds showed higher flavor dilution factors in the SE-SAFE method [16].

#### 3.2.2. Flavonoids

ATF is a rich source of flavonoids, and its methanolic extract has been reported to contain substantial amounts of flavonoids (1.21 mg QE per g DW) [4]. The major flavonoids identified in AT include quercetin (**73**), catechin (**69**), and epicatechin (**68**), all of which exhibit diverse biological activities. For instance, quercetin (**73**) has been reported to exhibit potent neuroprotective and antioxidant effects, while epicatechin demonstrates excellent anti-inflammatory activity [17]. Further analysis revealed that flavonoids are the primary phenolic compounds present in the ethyl acetate fraction of the AT ethanol extract [18]. In recent years, a series of structurally novel flavanol hybrids have also been discovered in AT, including flavanol–menthane conjugates, flavanol–fatty alcohol hybrids, and flavanol–monoterpenoid hybrids, which are believed to have potential applications in diabetes treatment [19,20,21]. Recent research indicates that the flavanol–fatty alcohol hybrids Tsaokoflavanols A1–J1 (**129**–**138**) exhibit inhibitory activity against HPL and may provide therapeutic benefits for obesity and related metabolic disorders [22]. Furthermore, the ultrasonic-assisted extraction and HPD300 macroporous resin purification process, optimized through one-factor experiments combined with response surface methodology, significantly improved the extraction efficiency of flavonoids from AT, achieving a yield of 3.33%, which is markedly higher than that reported in previous methods. During the purification process, flavonoids eluted with 20% and 30% ethanol exhibited high flavonoid purity (>90%) and remarkable antioxidant activity, surpassing that of vitamin C (Vc). Further metabolomic analysis revealed that the major components of this flavonoid-enriched fraction include epicatechin, isoquercitrin, astragalin, kaempferol-3-O-rutinoside, and procyanidin B2. These constituents were collectively responsible for the excellent in vitro antioxidant and *α*-glucosidase inhibitory activities of the ATEE [23]. To maximize the retention of flavonoids, it is essential to select appropriate extraction and purification methods and to optimize the process conditions accordingly.

#### 3.2.3. Diarylheptanoids

Diarylheptanoids are a class of natural products characterized by a distinctive structural motif, consisting of a seven-carbon aliphatic chain connecting two aromatic rings at the 1- and 7-positions. These substances are considered possible components for nutritional supplements and pharmaceuticals due to their varied biological activities, which include anti-inflammatory, antitumor, antioxidant, and neuroprotective properties [24]. Clinical studies have demonstrated that this class of compounds (curcumin) exhibits a favorable safety profile at daily doses of up to 12 g, although their systemic bioavailability remains relatively limited [25]. Notably, diarylheptanoids are considered characteristic constituents of AT [26]. In AT, diarylheptanoids are predominantly found in the fruits and leaves, among which linear diarylheptanoids represent the predominant structural type. Representative compounds include tsaokoarylone (**144**) and hannokinol (**174**) [26]. Linear diarylheptanoids accumulate predominantly in mature fruits, with concentrations peaking in fruits harvested in October and November, suggesting that their biosynthesis and accumulation are closely associated with fruit developmental stages. Furthermore, through co-expression network analysis and phylogenetic analysis, several candidate genes potentially involved in diarylheptanoid accumulation were identified, among which two key genes, AmT044854 and AmT007727, were verified to participate in the biosynthesis of linear diarylheptanoids. These findings suggest that the accumulation of diarylheptanoids in AT is developmentally regulated, providing molecular evidence for their potential as quality markers and functional components [26]. Nevertheless, further studies are required to ascertain the exact concentrations of these compounds and their biological effects.

#### 3.2.4. Phenolic Acids

As shown in Table S1, phenolic acids are predominantly distributed in ATF, but they have also been detected in the seeds, including vanillic acid (**209**), 3-O-methylgallic acid (**204**), 3,4-dihydroxybenzoic acid (**206**), and 3,4-dimethoxybenzoic acid (**203**). These phenolic acids are well known for their antioxidant and anti-inflammatory activities [27]. Emerging evidence suggests that the phenolic acids identified in AT exhibit potential antidiabetic, anti-obesity, and anti-Alzheimer’s effects [18,28,29], highlighting their potential applications in the prevention and management of chronic metabolic and neurodegenerative disorders. Furthermore, these compounds can serve as precursors for various bioactive substances, holding significant development value in the food industry, pharmaceuticals, cosmetics, and related engineering fields [30]. Regarding extraction methods, since phenolic acids in plant tissues often form relatively stable complexes with proteins or polysaccharides, the extraction efficiency of single-solvent systems is typically limited. In contrast, mixed systems of organic solvents and water have been demonstrated to improve the extraction efficiency of phenolic acids from AT, thereby facilitating subsequent isolation, characterization, and bioactivity evaluation [31].

#### 3.2.5. Terpenes and Steroids

Terpenoids identified in AT mainly comprise monoterpenes, sesquiterpenes, diterpenes, and bicyclononane derivatives, most of which have been isolated from its fruits. Among these, monoterpenes and sesquiterpenes are considered key components of AEO and fragrance. Notably, certain oxygen-containing monoterpenes, namely 4-indanecarbaldehyde (**271**), 5-indanecarbaldehyde (**270**), and trans- and cis-2,3,3a,7a-tetrahydro-1H-indene-4-carbaldehyde (**256**), have thus far been reported exclusively in AT samples originating from China and Vietnam and have not been identified in other species. These compounds may therefore serve as valuable chemotaxonomic markers for AT, independent of the region of origin [32]. Wang et al. were the first to identify isospongian diterpenoids in the leaves of AT; these compounds exhibit a variety of health benefits, including anticancer, anti-inflammatory, antiviral, antifungal, and antihypertensive effects [33]. Furthermore, bicyclononane is a unique chemical compound found in AT; this class of compounds forms a bicyclic core utilizing hexane and pentane rings as their basic skeletons [7]. The bicyclononane compounds isolated from ATF are also important bioactive substances.

Phytosterols are a class of bioactive compounds widely found in plants and are recognized for their beneficial effects on lipid metabolism and cardiovascular health [34]. Sitosterol (**274**) and daucosterol (**275**), isolated from ATF, are representative phytosterols. Among them, sitosterol has been extensively studied for its diverse health-promoting properties, including cardioprotective, antitumor, and glucose-regulating activities [35]. Additionally, sitosterol serves as an important precursor for the in vitro synthesis of vitamin D analogs, such as D_2_, D_4_, and D_5_ [36]. Likewise, accumulating evidence has demonstrated the antitumor, neuroprotective, and hypoglycemic effects of daucosterol [37]. Collectively, these compounds contribute to the health-promoting potential of AT and support its application in functional foods, nutraceuticals, and pharmaceutical products.

#### 3.2.6. Others

**Figure 2:**
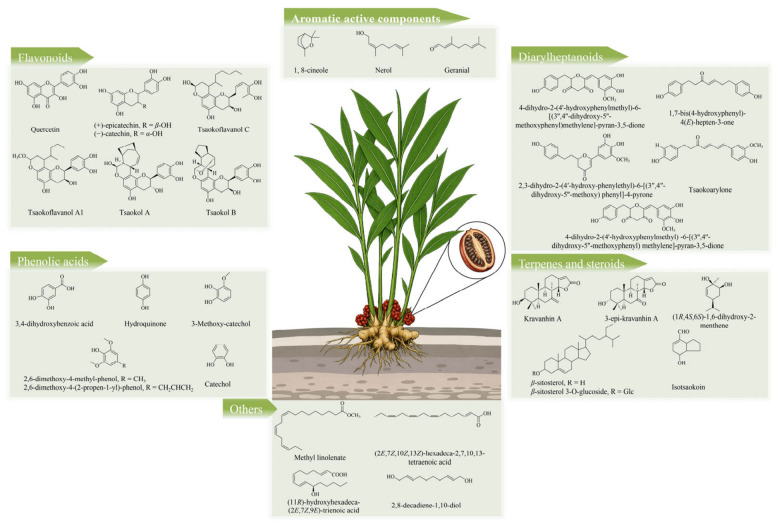
Structures of representative compounds in *Amomum tsaoko* Crevost & Lem.

Beyond the bioactive constituents discussed above, other types of compounds have been isolated from AT. These compounds encompass fatty acids, fatty aldehydes, fatty alcohols, aliphatic esters, pyrroles, and phenylethanoid glycosides. Among these constituents, the fatty acids (2*E*,7*Z*,10*Z*,13*Z*)-hexadeca-2,7,10,13-tetraenoic acid (**288**) and (2*E*,7*Z*)-tetradeca-2,7-dienoic acid (**289**), isolated from ATF, have demonstrated significant antibacterial, anti-obesity, and antioxidant activities [38]. Furthermore, the fatty acid (11*R*)-hydroxyhexadeca-(2*E*,7*Z*,9*E*)-trienoic acid (**290**), obtained from the ethanol extract of AT seeds, has been shown to inhibit sphingosine phosphatase SPHK1 or SPHK2 [28], suggesting its potential involvement in regulating lipid metabolism and associated signaling pathways. The structures of the representative compounds are shown in Figure 2.

## 4. Health Functions

Numerous studies have demonstrated that AT is rich in bioactive compounds that offer a broad spectrum of health benefits, including antibacterial, hypoglycemic, antioxidant, anti-inflammatory, anticancer, neuroprotective, anti-obesity, gastroprotective, and immunomodulatory effects, which will be briefly discussed below. The potential health benefits and bioactivities of AT are presented in Table 1.

### 4.1. Antibacterial Activity

In vitro studies have demonstrated that AT exhibits broad-spectrum antibacterial and antifungal activities. Its antibacterial activity is thought to be primarily attributed to AEO, which successfully inhibits both Gram-positive and Gram-negative bacteria as well as various fungi [39]. Among the tested microorganisms, AEO exhibits potent activity against *Staphylococcus aureus*, with a MIC of 0.20 mg/mL and an MBC ranging from 0.3–0.78 mg/mL [40], significantly outperforming other essential oils such as *Cinnamomum cassia* essential oil (2.5 mg/mL) [41] and *Zanthoxylum schinifolium* essential oil (10 mg/mL) [42]. Furthermore, 1,8-cineole, (E)-dec-2-enal, citral, *α*-pinene, and *α*-terpineol are considered the major contributors to the antimicrobial activity of AEO [40]. Mechanistic studies further demonstrated that AEO exerts rapid antibacterial effects against the foodborne bacterium *Escherichia coli* by disrupting membrane integrity and permeability, resulting in the leakage of intracellular nucleic acids and proteins [43]. In a mouse infection model, AEO protected animals from infections caused by *Staphylococcus aureus* or *Escherichia coli* in vivo, highlighting its potential for the management of bacterial infections, particularly those caused by multidrug-resistant pathogens [44]. It is worth noting that antimicrobial efficacy varies considerably for AEO obtained through different extraction methods. Compared to HD and SMFE extracts, the essential oil obtained via M-SFME exhibits stronger antimicrobial and antioxidant activities, primarily attributed to its higher proportion of oxygenated monoterpenes (55.8%) [15]. Furthermore, in vitro studies have also demonstrated that AEO exhibits pronounced antifungal activity against such fungi as *Botrytis cinerea*, *Aspergillus oryzae*, *Mucor*, and *Penicillium* [45].

In addition to AEO, ATE also exhibits notable antibacterial activity in vitro. Studies have shown that ATE inhibits bacterial growth by suppressing cellular respiration, mainly through disruption of the Krebs cycle during glucose metabolism [46]. Furthermore, by damaging bacterial cell wall integrity and morphology, it effectively inhibits *Listeria monocytogenes* and *Bacillus subtilis*, with MIC and MBC values of 1.25 mg/mL for both [47]. Beyond its direct antibacterial effects, ATE markedly suppresses biofilm formation, virulence factor production, and motility in various foodborne pathogens (such as *Staphylococcus aureus*, *Salmonella typhimurium*, and *Pseudomonas aeruginosa*) by interfering with bacterial quorum-sensing systems [48]. This demonstrates its potential value as a natural quorum-sensing inhibitor and anti-biofilm agent in controlling food spoilage and preventing bacterial infections. Interestingly, ATE exhibited remarkable stability under diverse environmental conditions. Its antibacterial activity showed high tolerance to changes in salt concentration, temperature, ultraviolet light, and pH, and exposure to salt, strong acids, and strong bases moderately enhanced its antibacterial activity. However, its antibacterial activity is relatively sensitive to high-sugar environments, where high-sugar environments reduce its antibacterial efficacy [49]. Antibacterial activity also varies considerably among extracts obtained using different solvents, with ethyl acetate extracts exhibiting the strongest antibacterial activity. For example, 95% ethanol and ethyl acetate extracts of ATF demonstrated excellent inhibitory activity against *Klebsiella pneumoniae* [38]. In addition, bicyclic nonane isotsaokoin (**262**), isolated from an ATFME, exhibited antifungal activity against Trichophyton mentagrophytes [50]. Nevertheless, current research primarily focuses on in vitro antimicrobial evaluations and preliminary investigations into mechanisms of action. Future studies should focus on identifying the key active components and clarifying their stability and safety in complex food systems or physiological environments to advance their practical application in food preservation and health-related fields.

### 4.2. Hypoglycemic Activity

**Figure 3:**
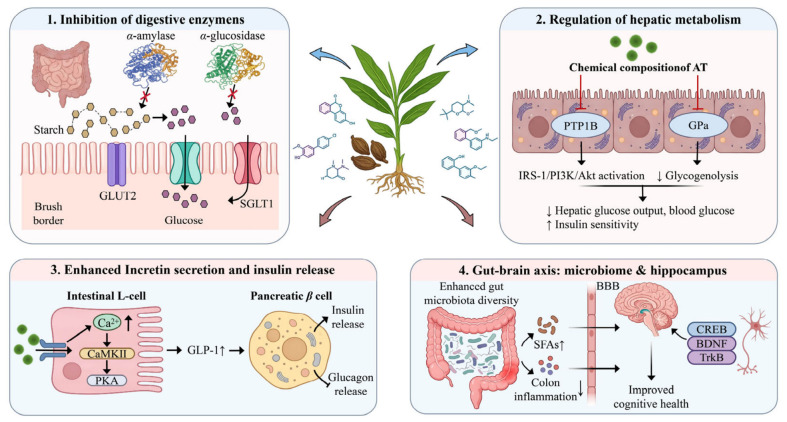
Hypoglycemic activity and its mechanisms of active compounds in *Amomum tsaoko* Crevost & Lem.

Diabetes is a major metabolic disorder associated with severe complications [51]. Many dietary natural products play an important role in the prevention and management of diabetes. As shown in Figure 3, AT has shown considerable potential for glycemic control and glucose metabolism regulation. In vitro studies showed that the ATME exhibits potent *α*-glucosidase inhibitory activity, with an IC_50_ value of 0.145 mg/mL, exceeding that of the clinical *α*-glucosidase inhibitor acarbose (IC_50_, 0.273 mg/mL) [52]. Similarly, both the AT water extract (seeds and pericarp) and the 50% ethanol–water extract exhibited significant inhibitory effects on *α*-glucosidase [53], while the water extract (seeds and pericarp) also effectively inhibited *α*-amylase activity [54]. Additionally, the extraction process using solvents of varying polarities markedly affects the antidiabetic activity of AT extracts. Fan et al. found that EF and BF exhibited superior *α*-glucosidase inhibitory activity compared to other fractions, with IC_50_ values of 20.14 ± 0.78 and 18.30 ± 0.42 μg/mL, respectively [18]. Evidence from in vivo studies further supports the antidiabetic potential of AT. In high-fat diet-induced diabetic mouse models, both the ATEE and ATME exhibited significant antidiabetic effects [4,18,52]. Mechanistic studies revealed that ATEE significantly reversed glucose metabolism disorders and cognitive deficits in mice with T2DM. These effects were associated with modulation of the gut microbiota, increased SCFA production, reduced inflammation in the hippocampus and colon, and activation of the CREB/BDNF/TrkB pathway [55]. These effects may be attributed to the phenolic compounds in AT, which may exert independent or synergistic actions on the aforementioned activities [54].

Bioactivity-guided fractionation and in vitro screening have identified numerous bioactive constituents responsible for the antidiabetic activity of AT. Several diarylheptanoids, including Tsaokopyranols A–M (**184**–**196**), exhibited potent α-glucosidase inhibitory activity, with IC_50_ values ranging from 59.4 to 116.5 μM, all showing stronger activity than acarbose (IC_50_ = 219.0 μM) [53]. Among these, Amomutsaokols H (**191**) and J (**193**) act as non-competitive α-glucosidase inhibitors, with K_i_ values of 18.5 and 213.0 μM, respectively [56]. Notably, 2-hydroxymusaitinerin A (**169**), a diarylheptanoid isolated from AT leaves, was identified as a novel inhibitor of GPa, PTP1B, and *α*-glucosidase, while platyphyllone (**179**) exhibited mixed-mode inhibition on *α*-glucosidase through both non-competitive and anti-competitive mechanisms [57]. Moreover, several flavonoids have also demonstrated promising antidiabetic activity in vitro. Among them, Tsaokols A (**140**) and B (**139**) showed notable *α*-glucosidase inhibitory activity, which was significantly stronger than acarbose [21]. Amomutsaokins A (**101**) and F (**106**) were identified as mixed-type α-glucosidase inhibitors, with K_i_ values of 36.2 and 24.4 μM, respectively [19]. Tsaokoflavanols A (**110**), B (**111**), F (**115**), and K (**120**) displayed dual inhibitory activity against α-glucosidase and PTP1B [20]. In addition, diterpenoids have also shown antidiabetic potential. Kravanhin A (**259**) and 3-epi-kravanhin A (**258**), isolated from AT leaves, significantly enhanced GLP-1 secretion in STC-1 cells via the Ca^2+^/CaMKII and PKA pathways [33]. Although AT extracts lower blood glucose in animal models, most isolated compounds have only been tested in enzyme- and cell-based assays. Future studies should validate their in vivo efficacy and evaluate pharmacokinetics and bioavailability.

### 4.3. Antioxidant Activity

Oxidative stress is associated with various pathological conditions, including cardiovascular disease, neurodegenerative diseases, cancer, and aging [58]. Therefore, antioxidant activity is essential for maintaining physiological homeostasis and can be enhanced by dietary antioxidants [59]. In in vitro experiments, the ATFME exhibited strong radical-scavenging activity against DPPH^.^ (IC_50_: 0.044 mg/mL) and ABTS^+^ (IC_50_: 0.040 mg/mL), indicating potent antioxidant activity [52]. Further studies suggest that the antioxidant constituents in AT are mainly concentrated in the polar fractions, particularly the methanol extract [60,61]. Different polar fractions of AT also exhibit antioxidant activity in vitro, although their relative effectiveness depends on the assay employed. Specifically, the EF showed the strongest DPPH^.^ scavenging activity, while the BF exhibited the greatest superoxide radical-scavenging and ferric-reducing capacities [62]. Similarly, the ethyl acetate fraction exhibited potent antioxidant activity with IC_50_ values of 0.17 and 0.07 mg/mL for DPPH^.^ and ABTS^+^, respectively, and a FRAP value of 546.10 mg VCE/g DW. This activity may be associated with the presence of phenolic hydroxyl groups or the synergistic effects of phenolic and flavonoid compounds [18]. Evidence from animal studies further supports the antioxidant potential of AT. Administration of ATFME significantly elevated SOD, GSH, and GSH-Px levels while reducing MDA and 8-ISO-PGF_2_*α* [52].

Chemical free radical-scavenging assays were used to further identify a number of antioxidant components in AT. Studies have demonstrated that AT polyphenols exhibit strong DPPH^.^ (IC_50_ = 42.46 μg/mL) and ABTS^+^ (IC_50_ = 85.47 μg/mL) scavenging activities, with antioxidant activity positively correlated with polyphenol content [63]. Further studies showed that the flavonoid quercetin (**73**) (inhibition rate > 80%), the phenolic acid 3,4-dihydroxybenzoic acid (**206**) (inhibition rate > 90%), and the diarylheptanoid CG-B (**142**) (inhibition rate > 79%) also exhibited strong DPPH scavenging activity at a concentration of 100 μg/mL, comparable to that of Vc at the same concentration [17,38,64]. Current antioxidant evidence relies largely on chemical radical-scavenging assays, which do not fully predict biological antioxidant efficacy. Future studies should assess whether these properties translate into physiological benefits in vivo.

### 4.4. Anti-Inflammatory Activity

**Figure 4:**
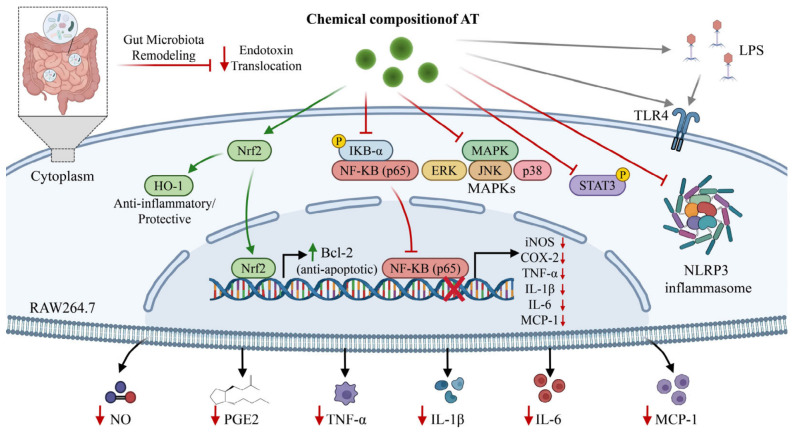
Anti-inflammatory activity and its mechanisms of active compounds in *Amomum tsaoko* Crevost & Lem.

Increasing evidence has highlighted the anti-inflammatory potential of AT extracts and their bioactive constituents. As shown in Figure 4, AT and its active components have demonstrated significant anti-inflammatory effects in both in vitro and in vivo studies. At the cellular level, the ATEE suppresses inflammatory responses in LPS-activated macrophages via Nrf2-dependent HO-1 expression and inhibition of NF-κB signaling [65]. Likewise, ATFME markedly inhibits NO production in LPS-stimulated BV2 microglia [66]. Mechanistic studies further showed that the ATFME reduces inflammation by inhibiting NO generation through the stimulation of the ROS/MAPK/Nrf2-mediated HO-1 signaling pathway [67]. In addition, AEO exhibited potent anti-inflammatory activity in LPS-stimulated macrophages. At non-cytotoxic concentrations (0–20 μg/mL), AEO reduced NO production and decreased the expression of TNF-*α*, IL-1*β*, IL-6, and MCP-1 in a dose-dependent manner. Concurrently, AEO effectively suppressed the overexpression of iNOS and COX-2 while reducing the levels of NF-κB*p*-p65 and MAPK*p*-ERK, suggesting that its anti-inflammatory activity is mediated by the inhibition of the NF-κB and MAPK signaling pathways [68]. Notably, evidence from animal studies further supports the anti-inflammatory potential of AT. In an LPS-induced mouse model of neuroinflammation, AEO improved cognitive performance and preserved neuronal integrity, upregulated the expression of the anti-apoptotic protein Bcl-2, and effectively alleviated the neuroinflammatory response. Multi-omics analyses further suggested involvement in the regulation of the MAPK signaling pathway, as well as the coordinated regulation of multiple metabolic pathways, including histidine metabolism, pantothenic acid and coenzyme A biosynthesis, and phenylalanine metabolism [69]. Recent studies suggest that AT alleviates ulcerative colitis through inhibiting STAT3 phosphorylation and suppressing necrotic apoptosis [70]. Additionally, AT flavonoids may ameliorate colitis by reshaping the gut microbiota, reducing endotoxin translocation, and thereby inhibiting the downstream TLR4/NF-κB/NLRP3 inflammatory signaling pathways [71].

Several anti-inflammatory compounds have now been identified in AT, with most of the evidence to date coming from cell-based experiments. In LPS-stimulated RAW 264.7 macrophages, the fatty acid methyl linolenate (**283**) inhibited NO production (IC_50_ = 61.2 μM) [72]. Two diarylheptanoids isolated from ATF, CG-A (**141**) and CG-B (**142**), also showed dose-dependent anti-inflammatory effects in vitro [73]. Further studies showed that the fatty alcohol compound 2,8-decadiene-1,10-diol (DDO) (**281**) suppresses NO and prostaglandin E_2_ production by downregulating the expression of iNOS and COX-2 and reduces the production of pro-inflammatory cytokines such as IL-6 and TNF-*α*. These effects are associated with the inactivation of MAPK signaling pathways, including ERK, JNK, and p38 MAPK, together with suppression of NF-κB signaling (including IκB-*α* degradation and NF-κB nuclear localization) [74]. Similarly, the flavonoids (+)-epicatechin (**68**) and (−)-catechin (**69**), as well as the terpenoid compound (1R,4S,6S)-1,6-dihydroxy-2-menthene (**254**), effectively reduced NO production in LPS-stimulated RAW 264.7 cells through inhibition of iNOS expression and inflammatory cytokine production. Among these, (+)-epicatechin (**68**) and (−)-catechin (**69**) exert anti-inflammatory effects mainly through inhibition of NF-κB nuclear translocation [75,76]. In summary, various extracts, essential oils, and isolated compounds from AT have demonstrated significant anti-inflammatory activity across multiple inflammatory models, primarily through modulation of oxidative stress and suppression of inflammatory signaling pathways.

### 4.5. Anticancer Activity

Cancer remains a major global health challenge. Natural products have attracted considerable attention as sources of anticancer agents because of their accessibility, suitability, and low cytotoxicity, and have been widely explored for cancer prevention and treatment [77]. Current evidence suggests that AT possesses promising antitumor activity in both in vitro and in vivo models. The AEO exhibits significant cytotoxicity against several human cancer cell lines, including HepG2, HeLa, and Bel-7402, in vitro. The strongest effect was observed against HepG2 cells (IC_50_ = 31.80 µg/mL), while its toxicity to normal cells, such as HUVEC and HL-7702, was low, suggesting selective anticancer activity. Mechanistically, AEO exerts its antitumor effects through apoptosis induction [78]. In addition to AEO, the ATEE also exhibits broad-spectrum antitumor activity. Studies have shown that the 95% ethanol extract and its ethyl acetate fraction exhibit potent cytotoxicity toward cervical cancer cells (HeLa), liver cancer cells (HepG-2 and SMMC-7721), and lung cancer cells (A549) [64]. Both cell-based and animal studies have further demonstrated that the ATEE suppresses ovarian tumor growth and reduces tumor-associated angiogenesis. Mechanistic analysis indicates that the *p*-STAT3/NF-κB signaling pathway forms a positive feedback circuit that promotes IL-6 and VEGF expression, while IL-6 and VEGF further activate this pathway, promoting tumor progression. The ATEE disrupts this cascade amplification effect by inducing endoplasmic reticulum stress, thereby suppressing angiogenesis and tumor progression [79].

In AT, several small-molecule constituents have shown cytotoxic and antiproliferative activities. Multiple fatty acids and diarylheptanoids isolated from ATF exhibited antiproliferative activity in the murine neuroblastoma cell line N2a. Among these compounds, tsaokoarylone (**144**) showed the strongest activity [80]. Additionally, hannokinol (**174**) and the flavonoid CG-B (**142**), isolated from the EF, showed significant cytotoxic action against HepG-2, SMMC-7721, HeLa, and A549 cell lines. CG-B was particularly active against SMMC-7721 cells (IC_50_ = 44.66 µg/mL); this activity even surpassed that of the positive control 5-fluorouracil [64]. At the same time, the flavanol hybrid tsaokoflavanol C (**112**) induced apoptosis and inhibited the proliferation of HepG2 cells [22]. However, current research is primarily conducted on various tumour cell lines in vitro. Future investigations should evaluate whether these cytotoxic effects can be reproduced in animal tumor models while minimizing toxicity toward normal tissues.

### 4.6. Neuroprotective Effects

Numerous studies have demonstrated that AT exhibits broad neuroprotective effects across multiple neurological disease models through multi-target mechanisms. Compared to other spices such as cinnamon, cardamom, black pepper, and white pepper, AT exhibited stronger anti-Alzheimer’s disease (AD) activity. Specifically, AEO demonstrated significant acetylcholinesterase inhibitory activity and improved behavioral deficits in an AlCl_3_-induced zebrafish dementia model, supporting its neuroprotective potential [81]. In a mouse model of pentylenetetrazol (PTZ)-induced seizures, the ethanol extract of ATF exerted antiepileptic and neuroprotective effects through antioxidant, anti-inflammatory, neurotransmitter-regulating, and energy metabolism-modulating activities [82]. In a mouse model of diabetes-induced depression, ATEE improved metabolic disorders and depressive-like behaviors, increased neuroendocrine hormone secretion, modulated microglial activation, and promoted neurogenesis. It also inhibited the expression of inflammation-related proteins such as HMGB1, TLR4, and NF-κB, thereby alleviating diabetes-associated depression, suggesting that the HMGB1/TLR4/NF-κB signaling axis may be a key mechanism underlying its effects [83]. Furthermore, in a mouse model of Parkinson’s disease induced by low-dose rotenone, AT flavonoids alleviated motor dysfunction and constipation, protected dopaminergic neurons, reduced inflammatory responses, and enhanced intestinal barrier function. Notably, their anti-Parkinson’s mechanism may also be associated with the regulation of gut microbiota [84].

Cell-based studies have indicated that the representative flavonoid quercetin showed significant neuroprotective effects against H_2_O_2_-induced injury in PC12 cells, significantly improving cell viability at a concentration of 50 μg/mL [17]. Furthermore, diarylheptanoids CG-A (**141**) and CG-B (**142**) significantly attenuated H_2_O_2_-induced cytotoxicity, with CG-A (141) exhibiting greater neuroprotective activity than CG-B (**142**), showing activity comparable to that of vitamin C at the tested concentration [73]. Another study reported that compounds isolated from ATF, including tsaokoic acid (**264**), tsaokoin (**263**), vanillin (**214**), and tsaokoarylone (**144**), have shown potential cognitive-enhancing and anti-Alzheimer’s disease activities [29]. Collectively, available evidence suggests that AT has promising neuroprotective potential, primarily by modulating oxidative stress and neuroinflammatory pathways. Future studies should confirm whether these effects can be replicated in more clinically relevant models and establish the long-term safety of AT supplementation.

### 4.7. Anti-Obesity Activity

ATE has significant anti-obesity effects. In diet-induced obese mice, the ATEE alleviated obesity-associated dyslipidemia as evidenced by reduced body weight gain, visceral adiposity, and dyslipidemia-related biomarkers [85]. Similarly, studies in ovariectomized mice demonstrated that the ATEE dose-dependently suppressed estrogen deficiency-induced weight gain and significantly reduced abnormal lipid accumulation in adipose tissue, the liver, and bone marrow, suggesting a beneficial role in hormone-related metabolic disorders [86]. Moreover, based on the results of network pharmacology analysis, AT contains several active compounds that are effective for treating obesity, with concentrations being relatively higher in AT harvested in September and October [87]. In addition, in HFD-induced NAFLD mice, AT effectively reduced lipid accumulation, oxidative stress, and inflammation while improving gut microbiota composition and lipid metabolism. Its protective effects are closely associated with the regulation of the gut–liver axis, suggesting potential applications in NAFLD prevention and management [88].

Among the isolated compounds, the flavanol–fatty alcohol hybrids Tsaokoflavanols A1 (**129**), B1 (**130**), E1 (**133**), and F1 (**134**) isolated from AT exhibited potent HPL inhibitory activity, with IC_50_ values ranging from 0.017 to 0.193 mM. Notably, Tsaokoflavanol A1 (**129**) exhibited stronger HPL inhibition than the positive control orlistat (IC_50_ = 0.067 mM) [22]. Similarly, (2*E*,7*Z*)-tetradeca-2,7-dienoic acid (**289**) and (*E*)-tetradec-2-enoic acid (**287**) also showed stronger lipase inhibitory activity than orlistat at a concentration of 50 μg/mL [38]. Furthermore, methyl linolenate (**283**) and catechol (**233**), identified from the ATEE via bioactivity-guided fractionation, displayed significant anti-adipogenic activity in the 3T3-L1 preadipocyte model. Both compounds reduced lipid accumulation in a dose-dependent manner and were more effective than other co-isolated constituents [89].

Mechanistically, current evidence suggests that the anti-obesity effects of AT are mediated through multiple complementary mechanisms, including inhibition of dietary lipid digestion via pancreatic lipase, suppression of adipocyte differentiation, attenuation of ectopic lipid accumulation, and modulation of the gut–liver axis. Among these, pancreatic lipase is the only molecular target that has been directly identified for AT extracts. Although methyl linolenate (**283**) and catechol (**233**) exhibit anti-adipogenic activity in 3T3-L1 preadipocytes, their effects on key adipogenic regulators, such as peroxisome proliferator-activated receptor γ (PPARγ) and CCAAT/enhancer-binding protein *α* (C/EBP*α*), remain unknown. Likewise, no studies have investigated whether AT promotes white adipose tissue browning or enhances lipolysis by regulating thermogenic markers (e.g., UCP1, PRDM16, and PGC-1*α*) or lipolytic enzymes (e.g., ATGL and HSL). Overall, current evidence supports the anti-obesity effects of AT mainly through reduced lipid digestion, suppression of adipogenesis, and improved lipid metabolism. Future studies should determine whether AT also modulates thermogenesis and lipolysis and identify the underlying molecular targets.

### 4.8. Gastrointestinal Protective Effects

Traditionally, AT has been used to alleviate gastrointestinal discomfort. Recent studies have provided growing evidence supporting its gastrointestinal benefits. Relevant studies have confirmed that its active components include essential oils, water extracts, and flavonoids. Diets containing AT significantly modulated the intestinal microbiota of zebrafish and tilapia, promoting beneficial microorganisms, suppressing potentially harmful bacteria, and improving gut morphology. These findings suggest that AT can improve gut health by regulating the gut microbiome and intestinal morphology, supporting its application as a natural feed additive in aquaculture [90]. Additionally, AEO dose-dependently alleviated loperamide-induced gastrointestinal hypomotility in rats. Non-targeted metabolomics indicated that AEO normalizes several dysregulated metabolite levels by modulating pathways such as primary bile acid biosynthesis. Microbiome-metabolite correlation analysis further revealed that AEO improved intestinal homeostasis and motility by regulating the gut microbiota structure and metabolic function [91].

Beyond AEO, AT water extracts and their flavonoid compounds have also shown promising laxative activity. In a mouse model of loperamide-induced constipation, medium- and high-dose AT aqueous extract (ATAE) (750 and 1000 mg/kg/day) significantly improved gastrointestinal transit and defecation parameters, confirming its laxative effect [92]. Recent studies have further confirmed that ATAE effectively alleviates loperamide-induced slow-transit constipation, with the bioactivity primarily attributed to the ethanol-soluble fraction of AT (ATES) rather than the ethanol precipitate of ATAE. Flavonoids are the primary active components of ATES, and the flavonoid fraction (ATTF) purified from ATES also exhibits pronounced laxative activity. Mechanistic investigations indicate that ATTF may alleviate constipation by modulating gut microbiota composition (e.g., *Lactobacillus* and *Bacillus*), regulating metabolites associated with serotonergic signaling pathways (such as PGJ2 and TXB2), and improving serotonergic synaptic pathway-related factors (such as 5-HT), thereby promoting intestinal motility and maintaining gut homeostasis [93].

Although AT supplementation beneficially modulates gut microbiota composition and improves intestinal homeostasis, its effect on specific pathobionts such as *Fusobacterium nucleatum* remains unexplored. *F. nucleatum* is a key intestinal pathobiont linked to gut barrier dysfunction, chronic inflammation, inflammatory bowel disease, and colorectal cancer [94]. AT supplementation has been shown to increase beneficial bacteria, suppress potentially harmful microorganisms, improve gut morphology, and regulate microbial metabolites linked to intestinal function. In addition, AT and its bioactive constituents exhibit broad-spectrum antimicrobial, anti-inflammatory, and barrier-protective activities, which may indirectly create an intestinal microenvironment unfavorable for the colonization or expansion of *F. nucleatum*. Future studies should assess whether AT alters intestinal *F. nucleatum* abundance by combining microbiome sequencing with targeted bacterial quantification.

### 4.9. Immunomodulatory Effects

Both in vitro and in vivo studies have demonstrated that the AT polysaccharide ATP-4 can enhance immune responses through direct activation of immune cells and indirect modulation of the gut microbiota. Subsequent studies revealed that the polysaccharide ATP-4e, generated through structural modification of ATP-4 while preserving the active domain AO-2, exhibits enhanced immunomodulatory activity relative to native ATP-4 [9]. In addition to polysaccharides, the ATSEE and its low-molecular-weight components also exhibit immunomodulatory effects. In vitro studies have shown that the ATSEE can effectively inhibit the activity of sphingosine phosphatase (SPHK1/2). Tsaokol A (**140**), further isolated from the extract, exhibited strong inhibitory effects on both SPHK1 and SPHK2, with inhibition rates of 59.75% and 25.40%, respectively. Furthermore, 8-hydroxy-2,6-dimethyl-1,6-octadien-3-one (**246**) exhibited significant inhibitory activity against SPHK2 (inhibition rate of 22.75%), outperforming the positive control [28]. These results suggest that the ATSEE and its bioactive small molecules may participate in immune regulation by modulating sphingosine metabolism-related pathways. Additionally, various phenolic compounds isolated from AT have demonstrated notable anti-complement activity in vitro. Related studies have shown that these compounds primarily exert their effects by inhibiting the classical and alternative pathways of the complement system. Among them, 1,7-bis(4-hydroxyphenyl)-4(E)-hepten-3-one (**183**) and hydroquinone (**232**) can interact with multiple key complement components, including C1q, C2, and C3, thereby regulating the complement cascade [95]. In summary, the immunomodulatory effects of AT involve immune cell activation, gut microbiota regulation, modulation of sphingosine metabolism, and inhibition of the complement system. These findings support the potential use of AT in immune-related functional foods or health products. However, its specific molecular mechanisms and in vivo actions require further study.

### 4.10. Others

Beyond the pharmacological activities described above, AT has also been reported to possess anti-atherosclerotic, antiviral, nephroprotective, anti-*Trichomonas vaginalis*, cholesterol-lowering, and anti-osteoporotic effects.

AT may exert anti-atherosclerotic effects through the regulation of lipid metabolism, attenuation of inflammation, and modulation of gut microbiota composition [96]. The available evidence indicates that the cardiovascular benefits of AT are currently supported primarily through indirect mechanisms. By improving hyperglycemia, obesity, dyslipidemia, oxidative stress, chronic inflammation, and gut microbiota dysbiosis, AT may reduce multiple cardiometabolic risk factors that contribute to the development of atherosclerosis. However, direct evidence demonstrating cardiovascular protection, such as vascular endothelial protection, improvement of vascular function, or attenuation of myocardial injury, remains limited. Therefore, future studies are warranted to determine whether AT exerts direct cardiovascular effects independent of its metabolic regulatory activities. Furthermore, AEO can interact with the SARS-CoV-2 spike protein RBD in aerosols, thereby preventing RBD–hACE2 binding and potentially interrupting viral transmission, thereby demonstrating significant potential for preventing aerosol transmission of the virus [97]. In a gentamicin-induced rat model, administration of AEO reduced serum urea and creatinine levels in the rats and improved pathological changes and oxidative stress in renal tissue. Concurrently, AEO alleviated inflammatory responses and apoptosis in the rats by regulating the MAPK pathway, indicating a nephroprotective effect of AEO [98]. Additionally, both AEO and geraniol (**38**) exhibited anti-T. vaginalis activity, which may result from damage to cell membranes and organelles [99]. In another study, AEO and polyphenol extracts reduced cholesterol levels by upregulating hepatic CYP7A1 expression, promoting bile acid excretion, and modulating gut microbiota involved in cholesterol metabolism, such as *Ruminococcaceae* [100]. Regarding anti-osteoporosis effects, Shim et al. demonstrated that ATEE significantly improved bone microarchitecture, bone mineral density, and bone volume fraction in an ovariectomized mouse model. This effect was primarily attributed to the inhibition of osteoclast differentiation. In vitro experiments confirmed that ATEE effectively inhibits osteoclast formation by downregulating the RANKL-induced NF-κB/Fos/NFATc1 signaling pathway, thereby suppressing bone resorption [86]. Despite the promising pharmacological activities reported for AT, current evidence is mainly based on cell and animal studies. Clinical validation remains limited, and future randomized controlled trials are needed to confirm its efficacy and safety in humans.

**Table 1:** Health functions and related mechanisms of *Amomum tsaoko* Crevost & Lem. (“↓”, decrease; “↑”, increase).

Bioactivities/Components	Assays	Testing Subjects	Effects/Mechanisms	References
Antibacterial activity				
AEO	In vitro	Agar disc diffusion assay, MIC	*Staphylococcus aureus* CCTCC AB91118 (MIC and MBC = 0.20 g/L)	[39]
AEO	In vitro	Agar disc diffusion assay, MIC, MBC	*Staphylococcus aureus* (MIC = 0.20 mg/mL, MBC = 0.39–0.78 mg/mL)	[40]
AEO	In vitro	Agar disk diffusion assay, MIC, MBC	*Escherichia coli* (MIC = 3.13, MBC = 6.25 mg/mL)	[43]
AEO	In vitro	MIC	Gram-positive and Gram-negative bacteria (MIC = 22.49 to 1438.91 μg/mL)	[44]
AEO	In vivo	Mice	Inhibiting *Escherichia coli*, *Staphylococcus aureus*	[44]
AEO	In vitro	MIC, MBC	Gram-positive and Gram-negative bacteria (MIC = 2.94–5.86 mg/mL)	[15]
ATE	In vitro	MIC, MBC	*Bacillus subtilis* and *Listeria monocytogenes* (MIC and MBC = 1.25 mg/mL)	[47]
ATFME	In vitro	Disk diffusion assay, MIC	*Staphylococcus aureus* (MIC = 1 mg/mL); *Salmonella typhimurium* (MIC = 2 mg/mL); *Pseudomonas aeruginosa* (MIC = 2 mg/mL)	[48]
95% ethanol and ethyl acetate fraction	In vitro	*Klebsiella pneumoniae*	Inhibiting *Klebsiella pneumoniae*	[38]
(2*E*,7*Z*,10*Z*,13*Z*)-hexadeca-2,7,10,13-tetraenoic acid	In vitro	*Klebsiella pneumoniae*	Inhibiting *Klebsiella pneumoniae* (inhibition rate > 99%)	[38]
(2*E*,7*Z*)-tetradeca-2,7-dienoic acid	In vitro	*Klebsiella pneumoniae*	Inhibiting *Klebsiella pneumoniae* (inhibition rate > 99%)	[38]
(*E*)-tetradec-2-enoic acid	In vitro	*Klebsiella pneumoniae*	Inhibiting *Klebsiella pneumoniae* (inhibition rate > 99%)	[38]
(*E*)-dodec-2-enoic acid	In vitro	*Klebsiella pneumoniae*	Inhibiting *Klebsiella pneumoniae* (inhibition rate > 99%)	[38]
Coronadiene	In vitro	*Klebsiella pneumoniae*	Inhibiting *Klebsiella pneumoniae* (inhibition rate > 99%)	[38]
Vanillic acid	In vitro	*Klebsiella pneumoniae*	Inhibiting *Klebsiella pneumoniae* (inhibition rate > 99%)	[38]
3,4-dihydroxybenzoic acid	In vitro	*Klebsiella pneumoniae*	Inhibiting *Klebsiella pneumoniae* (inhibition rate > 99%)	[38]
Isotsaokoin	In vitro	Agar disc diffusion assay	Inhibiting *Trichophyton mentagrophytes*	[50]
AEO	In vitro	*Escherichia coli*, *Staphylococcus albus*, *Bacillus subtilis*, *Penicillium*, *Mucor*, and *Aspergillus oryzae*	Inhibiting *Bacillus subtilis*, *Staphylococcus albus*, and *Escherichia coli*; inhibiting *Aspergillus oryzae*, *Mucor*, and *Penicillium*	[45]
ATE	In vitro	*Staphylococcus aureus*	Inhibiting bacterial growth, altering cell morphology and cell membrane structure, inhibiting cellular respiration and metabolism	[46]
ATE	In vitro	MIC	*Escherichia coli* and *Candida albicans* (MIC = 1.5 mg/mL); *Bacillus subtilis* and *Proteus vulgaris* (MIC = 3.0 mg/mL); *Aspergillus niger* (MIC = 6.0 mg/mL).	[49]
Hypoglycemic activity				
ATME	In vitro	*α*-glucosidase, *α*-amylase	Inhibiting *α*-glucosidase and *α*-amylase	[61]
EF	In vitro	*α*-glucosidase	Inhibiting *α*-glucosidase (IC_50_ = 20.14 ± 0.78 μg/mL)	[18]
BF	In vitro	*α*-glucosidase	Inhibiting *α*-glucosidase (IC_50_ = 18.30 ± 0.42 μg/mL)	[18]
EF	In vivo	HFD-induced diabetes mice	Fasting blood glucose ↓, glucose tolerance ↑	[18]
ATME	In vitro	*α*-glucosidase	Inhibiting *α*-glucosidase (IC_50_ = 0.154 mg/mL)	[52]
ATME	In vivo	STZ-induced diabetic mice	Fasting blood glucose, area under the curve of the oral glucose tolerance test, HOMA-IR ↓, HOMA-*β* ↑	[52]
50% ethanol–water extract	In vitro	*α*-glucosidase	Inhibiting *α*-glucosidase (IC_50_ = 38.6 μg/mL)	[53]
Tsaokopyranol E	In vitro	*α*-glucosidase	Inhibiting *α*-glucosidase (IC_50_ < 100 μM)	[53]
Tsaokopyranol H	In vitro	*α*-glucosidase	Inhibiting *α*-glucosidase (IC_50_ < 100 μM)	[53]
Tsaokopyranol I	In vitro	*α*-glucosidase	Inhibiting *α*-glucosidase (IC_50_ < 100 μM)	[53]
Tsaokopyranol J	In vitro	*α*-glucosidase	Inhibiting *α*-glucosidase (IC_50_ < 100 μM)	[53]
Tsaokopyranol K	In vitro	*α*-glucosidase	Inhibiting *α*-glucosidase (IC_50_ < 100 μM)	[53]
phaeoheptanoxide	In vitro	*α*-glucosidase	Inhibiting *α*-glucosidase (IC_50_ < 100 μM)	[53]
AT aqueous extracts	In vitro	*α*-glucosidase, *α*-amylase	Inhibiting *α*-amylase and *α*-glucosidase	[54]
ATE	In vivo	HFD and STZ-induced T2DM mice	Glucose tolerance, SOD ↑, FBG, insulin, MDA ↓, maintaining pancreatic structure and function	[4]
ATE	In vitro	*α*-glucosidase, *α*-amylase	Inhibiting *α*-glucosidase (IC_50_ = 1.76 mg/mL) and *α*-amylase (IC_50_ = 14.23 mg/mL)	[4]
ATEE	In vivo	HFD and STZ-induced T2DM mice	The expression of proteins in the CREB/BDNF/TrkB pathway, SCFA ↑, inflammatory responses in the hippocampus, the loss of colonic tight junction proteins, levels of colonic inflammatory factors ↓, reshaping the gut microbiota	[55]
2-Hydroxymusaitinerin A	In vitro	Enzyme inhibition assay	Inhibiting GPa (99.0%), PTP1B (59.4%), and *α*-glucosidase (55.9%)	[55]
Platyphyllone	In vitro	*α*-glucosidase	Inhibiting *α*-glucosidase via non-competitive and competitive mechanisms (IC_50_ = 25.8 μM)	[55]
Tsaokol A	In vitro	*α*-glucosidase	Inhibiting *α*-glucosidase (IC_50_ = 18.8 μmol/L)	[21]
Tsaokol B	In vitro	*α*-glucosidase	Inhibiting *α*-glucosidase (IC_50_ = 38.6 μmol/L)	[21]
proanthocyanidin A-2	In vitro	Enzyme inhibition assay	Inhibiting PTP1B (IC_50_ = 201.45 μM); Inhibiting *α*-glucosidase (IC_50_ = 3.73 μM)	[19]
Amomutsaokin B	In vitro	Enzyme inhibition assay	Inhibiting PTP1B (IC_50_ = 314.00 μM); Inhibiting *α*-glucosidase (IC_50_ = 73.05 ± 4.60 μM)	[19]
Amomutsaokin C	In vitro	Enzyme inhibition assay	Inhibiting PTP1B (IC_50_ = 266.31 μM); Inhibiting *α*-glucosidase (IC_50_ = 76.23 ± 0.57 μM)	[19]
Amomutsaokin F	In vitro	Enzyme inhibition assay	Inhibiting PTP1B (IC_50_ = 317.51 μM); Inhibiting *α*-glucosidase (IC_50_ = 29.50 ± 9.05 μM)	[19]
flavanocoumarin	In vitro	Enzyme inhibition assay	Inhibiting PTP1B (IC_50_ = 285.62 μM)	[19]
Amomutsaokin A	In vitro	Enzyme inhibition assay	Inhibiting *α*-glucosidase (IC_50_ = 61.45 ± 12.80 μM)	[19]
Amomutsaokin E	In vitro	Enzyme inhibition assay	Inhibiting *α*-glucosidase (IC_50_ = 48.45 ± 0.07 μM)	[19]
Amomutsaokin G	In vitro	Enzyme inhibition assay	Inhibiting *α*-glucosidase (IC_50_ = 34.94 ± 3.08 μM)	[19]
Amomutsaokin H	In vitro	Enzyme inhibition assay	Inhibiting *α*-glucosidase (IC_50_ = 30.70 ± 1.13 μM)	[19]
(+)-afzelechin	In vitro	Enzyme inhibition assay	Inhibiting *α*-glucosidase (IC_50_ = 44.10 ± 2.55 μM)	[19]
sappanone B	In vitro	Enzyme inhibition assay	Inhibiting *α*-glucosidase (IC_50_ = 64.95 ± 7.71)	[19]
Brazilin	In vitro	Enzyme inhibition assay	Inhibiting *α*-glucosidase (IC_50_ = 34.40 ± 1.41 μM)	[19]
Tsaokoflavanols A, B, F, K, R	In vitro	Enzyme inhibition assay	Inhibiting *α*-glucosidase (IC_50_ = 5.2–9.0 μM)	[20]
Tsaokoflavanols F, J, K, L, S	In vitro	Enzyme inhibition assay	Selectively inhibiting PTP1B/TCPTP (IC_50_ = 56.4–80.4 μM)	[20]
ATE	In vivo	*db/db* mice	The random blood glucose and fasting blood glucose ↓	[20]
*Kravanhin C*	In vitro	Enzyme inhibition assay	Inflammation, oxidative stress, and insulin resistance ↓	[33]
3-epi-kravanhin A, kravanhin A	In vitro	STC-1 cells	GLP-1 secretion in STC-1 cells ↑	[33]
Antioxidant activity				
ATFME	In vitro	In vitro antioxidant assays	Scavenging DPPH^.^ and ABTS^+.^	[52]
ATFME	In vivo	D-galactose plus HFD induced oxidative damage mouse	SOD, GSH, GSH-Px ↑, MDA and 8-ISO-PGF_2_*α* ↓	[52]
ATME	In vitro	In vitro antioxidant assays	Scavenging DPPH^.^	[61]
ATME	In vivo	Mice	Plasma TBARS ↓	[61]
EF	In vitro	In vitro antioxidant assays	Scavenging DPPH^.^ (IC_50_ = 0.17 ± 0.01 mg/mL), ABTS^+.^ (IC_50_ = 0.07 ± 0.01 mg/mL); FRAP (546.10 ± 6.61 mg VCE/g DW)	[18]
*Quercetin*	In vitro	In vitro antioxidant assays	Scavenging DPPH^.^ (inhibition rate > 80% at a concentration of 100 mg/mL)	[17]
(2*E*,7*Z*,10*Z*,13*Z*)-hexadeca-2,7,10,13-tetraenoic acid	In vitro	In vitro antioxidant assays	Scavenging DPPH^.^ (inhibition rate = 77.08% at a concentration of 100 mg/mL)	[38]
3,4-dihydroxybenzoic acid	In vitro	In vitro antioxidant assays	Scavenging DPPH^.^ (inhibition rate > 90% at a concentration of 100 mg/mL)	[38]
95% ethanol extract and ethyl acetate fraction	In vitro	In vitro antioxidant assays	Scavenging DPPH^.^ (inhibition rate > 90% at a concentration of 200 mg/mL)	[64]
4-dihydro-2-(4′-hydroxy-phenylmethyl)-6- [(3″,4″-dihydroxy-5″-methoxyphenyl) methylene]-pyran-3,5- dione	In vitro	In vitro antioxidant assays	Scavenging DPPH^.^ (inhibition rate > 60% at a concentration of 80 g/mL); scavenging DPPH^.^ (inhibition rate = 79.04%)	[64]
2,3-dihydro-2-(4′-hydroxy-phenylethyl)-6- [(3″,4″-dihydroxy-5″- methoxy) phenyl]-4-pyrone	In vitro	In vitro antioxidant assays	Scavenging DPPH^.^ (inhibition rate = 58.55% at a concentration of 100 g/mL)	[64]
AEO	In vitro	In vitro antioxidant assays	Scavenging DPPH^.^ (IC_50_ = 5.27 mg/mL); the *β*-carotene/linoleic acid bleaching assay (IC_50_ = 0.63 mg/mL)	[15]
3,4-dihydroxybenzoic acid	In vitro	In vitro antioxidant assays	Scavenging DPPH^.^ (inhibition rate > 90% at a concentration of 100 μg/mL)	[38]
ATE	In vitro	In vitro antioxidant assays	Scavenging ABTS^+.^ (IC_50_ = 3.49 mg mL^−1^); ORAC = 34,276.57 μM TE/100 g DW; FRAP = 207.42 μM Fe^2+^ per g DW	[4]
EF	In vitro	In vitro antioxidant assays	Scavenging DPPH^.^ (99.82%)	[62]
BF	In vitro	In vitro antioxidant assays	Scavenging DPPH^.^ (91.79%); scavenging ABTS^+.^ (74.65%); reducing Fe^3+^ to Fe^2+^	[62]
AT polyphenols	In vitro	In vitro antioxidant assays	Scavenging DPPH^.^ (IC_50_ = 42.46 μg/mL); scavenging ABTS^+.^ (IC_50_ = 85.47 μg/mL)	[63]
Anti-inflammatory activity				
ATEE	In vitro	RAW 264.7 macrophages	Release of pro-inflammatory mediators, phosphorylation and degradation of IκB-*α*, the nuclear translocation of NF-κB p65 ↓	[65]
ATFME	In vitro	BV2 microglia	NO ↓	[66]
ATME	In vitro	RAW 264.7 murine macrophage cell line	iNOS expression ↓, HO-1 expression, Nrf2 expression, nuclear accumulation and the binding of Nrf2 to ARE ↑	[67]
ATME	In vivo	LPS-induced murine model of sepsis	Serum NO levels, hepatic iNOS expression ↓, HO-1 expression and survival rate ↑	[67]
AEO	In vitro	RAW 264.7 macrophages	NO, the expression levels of TNF-*α*, IL-1*β*, IL-6, MCP-1, iNOS, COX-2, NF-κB *p*-p65, and *p*-ERK ↓	[68]
AEO	In vivo	LPS-induced neuroinflammation mice	Bcl-2 expression ↑, the neuroinflammatory response ↓, improving cognitive function in LPS-induced neuroinflammatory mice, maintaining neuronal structural integrity	[69]
AT	In vitro	HT-29 cell line	Cell morphology and viability ↑, the phosphorylation of RIPK1, RIPK3, and MLKL ↓	[70]
AT	In vivo	DSS-induced ulcerative colitis injury mice	Expression of tight junction proteins ↑, weight loss, disease activity indices, distribution and expression of phosphorylated RIPK3 and MLKL ↓, improving intestinal histopathology	[70]
AT flavonoids	In vivo	Ulcerative coliti mice	Serum LPS, activation of the colonic TLR4/NF-κB/NLRP3 signaling pathway, *Escherichia*, *Shigella*, *Colidextribacter*, and *Oscillibacter* ↓, mRNA expression of tight junction proteins, *Akkermansia*, *Bifidobacterium*, unclassified_f__Atopobiaceae ↑, Improving body weight, disease activity index scores, and colon length in ulcerative coliti mice, alleviating colonic tissue damage	[71]
Amotsaokonal B	In vitro	RAW 264.7 macrophages	Inhibiting NO production (IC_50_ = 94.8 μM)	[72]
*Methyl linolenate*	In vitro	RAW 264.7 macrophages	Inhibiting NO production (IC_50_ = 61.2 μM)	[72]
CG-A	In vitro	RAW 264.7 macrophages	Inhibiting NO production (60.46 ± 0.23%)	[73]
CG-B	In vitro	RAW 264.7 macrophages	Inhibiting NO production (48.62 ± 0.38%)	[73]
2,8-decadiene-1,10-diol	In vitro	RAW 264.7 macrophages	Expression of inducible nitric oxide synthase and cyclooxygenase-2, NO and prostaglandin E2, pro-inflammatory cytokines such as IL-6 and TNF-*α* ↓	[74]
(+)-epicatechin	In vitro	RAW 264.7 cells	iNOS expression, inflammatory cytokines such as TNF-*α*, IL-1*β*, and IL-10, NO, nuclear localization of NF-κB ↓	[75]
(−)-catechin	In vitro	RAW 264.7 cells	iNOS expression, inflammatory cytokines such as TNF-*α*, IL-1*β*, and IL-10, NO, nuclear localization of NF-κB ↓	[75]
(1*R*,4*S*,6*S*)-1,6-dihydroxy-2-menthene	In vitro	RAW 264.7 cells	iNOS expression, NO ↓	[76]
Anticancer activity				
AEO	In vitro	HepG2, Bel-7402, Hela, A549, SGC -7901, PC-3 cells	Inhibiting growth of HepG2 (IC_50_ = 31.80 ± 1.18 μg/mL), HeLa, Bel-7402, A549, SGC-7901 and PC-3 cells	[78]
95% ethanol extract and ethyl acetate fraction	In vitro	HepG-2, SMMC-7721, HeLa, A549 cells	Inhibiting growth of HepG-2, SMMC-7721 (71.4%), HeLa, A549 cells	[64]
95% ethanol extract and petroleum ether fraction	In vitro	HepG-2, SMMC-7721, HeLa, A549 cells	Inhibiting growth of HepG-2, Hela, A549 cells	[64]
Isotsaokoin	In vitro	HepG-2, SMMC-7721, HeLa, A549 cells	Inhibiting growth of Hela (inhibition rate > 50%), HepG-2, SMMC-7721, A549 cells	[64]
Hannokinol	In vitro	HepG-2, SMMC-7721, HeLa, A549 cells	Inhibiting growth of A549 (65.9%) and HepG-2 (66.7%)	[64]
2,3-dihydro-2-(4′-hydroxy-phenylethyl)-6-[(3′′,4′’-dihydroxy-5′′-methoxy) phenyl]-4-pyrone	In vitro	HepG-2, SMMC-7721, HeLa, A549 cells	Inhibiting growth of A549 (70.03%)	[64]
4-dihydro-2-(4′-hydroxyphenylmethyl)-6-[(3′′,4′’-dihydroxy-5′′-methoxyphenyl) methylene]-pyran-3,5-dione	In vitro	HepG-2, SMMC-7721, HeLa, A549 cells	Inhibiting growth of SMMC-7721 (73.4%) and HepG-2 (68.3%)	[64]
ATEE	In vivo	BALB/c nude mice	Inhibiting tumor growth and angiogenesis	[79]
ATEE	In vitro	SKOV3, HUVEC cells	The *p*-STAT3/NF-κB positive feedback loop, IL-6, VEGF, migration, invasion, and tubulogenesis of vascular endothelial cells ↓	[79]
(2*E*,6*E*)-8-hydroxy-2,6-dimethyl-2,6-octadienal	In vitro	Mouse neuroblastoma cell line N2a	Inhibiting tumor proliferative (IC_50_ = 82 ± 2 μM)	[80]
Tsaokoarylone	In vitro	Mouse neuroblastoma cell line N2a	Inhibiting tumor proliferative (IC_50_ = 46 ± 7 μM)	[80]
(2*E*,8*E*)-10-hydroxy-decadienal	In vitro	Mouse neuroblastoma cell line N2a	Inhibiting tumor proliferative (IC_50_ = 52 ± 2 μM)	[80]
Tsaokoflavanol C	In vitro	HepG2 cells	Inducing HepG2 cells apoptosis (CC_50_ = 14.96 ± 0.62 Mm)	[22]
Neuroprotective effects				
AEO	In vivo	AlCl_3_-induced dementia in zebrafish	Improving the behavioral deficits in AlCl_3_-induced dementia zebrafish	[81]
AEO	In vitro	AChE	Inhibiting AChE (IC_50_ = 62.3 ± 11.0 μg/mL)	[81]
3-carene	In vitro	AChE	Inhibiting AChE (IC_50_ = 1.73 μg/mL)	[81]
*α*-pinene	In vitro	AChE	Inhibiting AChE (IC_50_ = 2.66 μg/mL)	[81]
*β*-pinene	In vitro	AChE	Inhibiting AChE (IC_50_ = 14.75 μg/mL)	[81]
AT fruit ethanol extract	In vivo	PTZ-induced seizure in mice	GABA, glutamate, and dopamine levels, Ca^2+^-ATPase and Na^+^-K^+^-ATPase activity ↑, frequency and duration of seizures, NF-κB, IL-1*β*, TLR-4, TNF-*α*, and COX-2 mRNA expression ↓	[82]
ATEE	In vivo	diabetic depression mice	Neurotransmitter levels, HMGB1, TLR4, and NF-κB proteins expression ↓, the secretion of neuroendocrine hormones ↑, improving glucose and lipid metabolism, the activation of microglia and the intensity of neurogenic immunofluorescence, alleviating depression-like behavior	[83]
AT flavonoids	In vivo	Rotenone-induced PD mouse	Dopaminergic neuron loss and inflammatory gene expression (TNF-α, IL-1β, IL-6, COX-2, and MCP-1) ↓, intestinal barrier-related gene expression (Muc-2, ZO-1, Occludin, Claudin-3, and Claudin-4) ↑, improving motor dysfunction and constipation symptoms, reversing rotenone-induced gut dysbiosis.	[84]
Quercetin	In vitro	PC-12 cells	Protecting PC-12 cells (survival rate = 78.9%)	[17]
EF	In vitro	PC-12 cells	Protecting PC-12 cells	[17]
Daucosterol	In vitro	PC-12 cells	Protecting PC-12 cells (survival rate = 75.6%)	[17]
Epicatechin	In vitro	PC-12 cells	Protecting PC-12 cells (survival rate = 70.4%)	[17]
Quercetin-7-O-*β*-glucoside	In vitro	PC-12 cells	Protecting PC-12 cells (survival rate = 68.1%)	[17]
Quercetin-3-O-*β*-glucoside	In vitro	PC-12 cells	Protecting PC-12 cells (survival rate = 68.1%)	[17]
Meso-hannokinol	In vitro	PC-12 cells	Protecting PC-12 cells (survival rate = 63.8%)	[17]
CG-A	In vitro	PC-12 cells	Protecting PC-12 cells (survival rate = 80.34 ± 1.78%)	[73]
CG-B	In vitro	PC-12 cells	Protecting PC-12 cells (survival rate = 69.82 ± 1.57%)	[73]
Tsaokoic acid	In vitro	AChE	Inhibiting AChE (IC_50_ = 32.78 μM)	[29]
Tsaokoin	In vitro	AChE	Inhibiting AChE (IC_50_ = 41.70 μM)	[29]
Vanillin	In vitro	AChE	Inhibiting AChE (IC_50_ = 39.25 μM)	[29]
Tsaokoarylone	In vitro	AChE	Inhibiting AChE (IC_50_ = 31.13 μM)	[29]
Anti-obesity activity				
ATEE	In vivo	C57BL/6 mice fed HCD	Body weight gain, visceral fat accumulation, subcutaneous fat accumulation, adipocyte size, plasmaTC and TG, LDL cholesterol, atherogenic index, cardiac risk factor, hepatic TC and TG content, hepatic lipid droplet accumulation ↓, HDL cholesterol ↑	[85]
ATEE	In vivo	ovariectomy mice	Weight gain, fat accumulation, osteoclast differentiation ↓, preventing ovariectomy-induced deterioration of bone density and trabecular bone microstructure	[86]
AT aqueous extract	In vivo	HFD-induced NAFLD mice	Weight gain, blood glucose, TG and cholesterol levels in serum and hepatic, pro-inflammatory cytokines (TNF-*α*, IL-6, and IL-1*β*) expression, hepatic lipogenesis genes (such as Ppar-*γ* and Fatp) expression ↓, hepatic antioxidant capacity, beneficial bacterial phyla abundance ↑, reversing HFD-induced dysbiosis, restoring microbial community diversity	[88]
Tsaokoflavanol A1	In vitro	HPL	Inhibiting HPL (IC_50_ = 0.017 mM,)	[22]
Tsaokoflavanol B1, D1, E1, F1, G1, I1	In vitro	HPL	Inhibiting HPL (IC_50_ = 0.091–0.483 mM)	[22]
(2*E*,7*Z*)-tetradeca-2,7-dienoic acid	In vitro	lipase	Inhibiting lipase (61.56%)	[38]
(*E*)- tetradec-2-enoic acid	In vitro	lipase	Inhibiting lipase (59.37%)	[38]
Methyl linolenate	In vitro	3T3-L1 cells	Lipid accumulation in 3T3-L1 adipocytes ↓	[89]
Catechol	In vitro	3T3-L1 cells	lipid accumulation in 3T3-L1 adipocytes ↓	[89]
Gastrointestinal protective effects				
AT Powder	In vivo	Zebrafish, tilapia	Beneficial bacteria, length of intestinal villi ↑, harmful bacteria ↓	[90]
AT Total Flavonoids	In vivo	Loperamide-induced constipation mice	Dominant commensals (such as *Lachnospiraceae*) ↓, *Lactobacillus* and *Bacillus*, 5-HT, the mRNA expression of 5-HT_2_A, PLA_2_, and COX2, TRPA1 and MLC3 expression ↑	[93]
AEO	In vivo	Loperamide hydrochloride-induced gastrointestinal motility inhibition rats	Restoring the abundance of the *Firmicutes* and *Verrucomicrobia* phyla, regulating metabolic pathways such as primary bile acid biosynthesis	[91]
AT aqueous extract	In vivo	Loperamide-induced constipation mice	First black faeces time ↓, the fecal water content, fecal weight and the number of defecations in 6 h, the intestinal transit ratio ↑	[92]
Immunomodulatory effects				
ATP-4	In vivo	CTX-induced bone marrow suppression mouse	Activating immune cells and regulating the gut microbiota	[9]
ATP-4	In vitro	RAW264.7 Cells	Activating immune cells and regulating the gut microbiota	[9]
Tsaokol A	In vitro	SPHK1/2	Inhibiting SPHK1 (40%), Inhibiting SPHK2 (70%)	[28]
8-hydroxy-2,6-dimethyl-1,6-octadien-3-one	In vitro	SPHK1/2	Inhibiting SPHK2 (70%)	[28]
1,7-bis(4-hydroxyphenyl)-4(*E*)-hepten-3-one	In vitro	Antibodies specific to complement components	Inhibiting the complement system components C1q, C2, C3, C4, C5, and C9	[95]
hydroquinone	In vitro	Antibodies specific to complement components	Inhibiting the complement system components C1q, C2, C3, C4, C5, and C9	[95]
Anti-atherosclerotic				
ATE	In vivo	atherosclerosis mice	TC, LDL, inflammatory responses in the aorta and liver ↓, regulating the gut microbiota, alleviating oxidative stress	[96]
Nephroprotective				
AEO	In vivo	gentamicin-induced acute kidney injury rats	Serum urea and creatinine levels, inflammatory responses, apoptosis ↓, improving renal histopathological changes and oxidative stress	[98]
Anti-*Trichomonas vaginalis*				
AEO	In vitro	*Trichomonas vaginalis* (Tv1, Tv2)	Damaging the cell membrane and organelles, *T. vaginalis* isolate Tv1 (MLC = 44.97 mg/mL, IC_50_ = 22.49 mg/mL), *T. vaginalis* isolate Tv2 (MLC = 89.93 mg/mL, IC_50_ = 44.97 mg/mL)	[99]
Geraniol	In vitro	*Trichomonas vaginalis* (Tv1, Tv2)	Damaging the cell membrane and organelles, *T. vaginalis* isolate Tv1 (MLC = 342.96 mg/mL, IC_50_ = 171.48 mg/mL); *T. vaginalis* isolate Tv2 (MLC = 342.96 mg/mL, IC_50_ = 171.48 mg/mL)	[99]
Cholesterol-lowering				
AT polyphenol extract	In vivo	Male Golden Syrian hamsters	Excretion of total acidic sterols, proliferation of the genus *Ruminococcus_2* ↑, plasma TC, the growth of *Allobaculum* and *Desulfovibrio* ↓	[100]
AEO	In vivo	Male Golden Syrian hamsters	Excretion of total acidic sterols, proliferation of the genus *Ruminococcus_2* ↑, plasma TC, the growth of *Allobaculum* and *Desulfovibrio* ↓	[100]
Anti-osteoporotic				
ATEE	In vivo	Ovariectomized mice	Weight gain, fat accumulation, osteoclast differentiation ↓, preventing deterioration of bone density and trabecular microstructure	[86]

## 5. Relationships Between Structure and Function

### 5.1. Structure–Function Relationship of Diarylheptanoids

Most bioactive diarylheptanoids identified in AT possess a linear skeleton [26]. Studies have shown that the introduction of fluorine and/or methoxy groups onto one or both aromatic rings of the linear di-aryl-heptane backbone markedly reduces cytotoxicity while largely preserving topoisomerase inhibitory activity, thereby improving the safety profile of these compounds [101]. Regarding antioxidant activity, the introduction of a catechol structure significantly enhances the antioxidant capacity of linear diarylheptanoids. At the same time, antioxidant potency is further affected by steric hindrance around the substitution site and the degree of unsaturation of the heptane chain [102]. Furthermore, the α-glucosidase inhibition is strongly influenced by the skeletal structure and the distribution of functional groups. Compounds featuring a linear 1,7-diarylheptane skeleton, a carbonyl group, and multiple free phenolic hydroxyl groups, such as platyphyllone (**179**), exhibit significantly enhanced inhibitory activity, as their hydroxyl and carbonyl groups facilitate stable hydrogen–bond interactions with the enzyme active site. In contrast, cyclized structures or highly substituted diarylheptanoid derivatives generally show weaker α-glucosidase inhibition due to conformational constraints or the shielding of key functional groups [57]. Further systematic analysis of Tsaokopyranols A–M (**184**–**196**) and Amomutsaokols A–K (**146**–**156**) indicates that hydroxylation modifications on the tetrahydrofuran ring are favorable for biological activity, with the configuration of the C-5 hydroxyl group on the C7 chain being a key factor influencing this activity. Additionally, the presence of two adjacent hydroxyl groups on the benzene ring is associated with enhanced activity, whereas acylation of the benzene ring and an increase in the number of methoxy groups both tend to diminish activity. Notably, the presence of a conjugated double bond may adversely affect activity, and the introduction of adjacent hydroxyl groups at the C-7 and C-8 positions appears to provide little or no benefit to activity [53,56].

Pharmacokinetic information on diarylheptanoids from AT is currently unavailable. However, the representative diarylheptanoid curcumin has been reported to exhibit poor oral bioavailability because of limited absorption, rapid metabolism, and rapid systemic elimination, highlighting pharmacokinetic limitations that may hinder the further development of this class of compounds [24,103]. Therefore, systematic pharmacokinetic evaluation of AT diarylheptanoids is warranted to support their future application as functional food ingredients and therapeutic candidates.

### 5.2. Structure–Function Relationship of Flavanol Hybrids

Existing studies indicate that the enzyme-inhibitory activity of flavanols and their hybrid derivatives is strongly influenced by their molecular aggregation form, substitution position, side-chain structure, and spatial conformation. Specifically, flavanol dimers generally display stronger *α*-glucosidase inhibitory activity than their monomeric counterparts. For flavanol–menthane conjugates, derivatives with menthane attached at the C-8 position show slightly stronger PTP1B inhibitory activity compared to compounds with menthane attached at the C-6 position, suggesting that the linkage position of the menthane moiety affects activity [19]. For flavanol–fatty alcohol hybrids, side-chain architecture plays a critical role in target enzyme selectivity. The study found that longer side chains enhanced PTP1B inhibition, while having minimal impact on *α*-glucosidase. Molecular docking further indicated that the hemiacetal hydroxyl group, the 3,4-dihydroxyphenyl group, and the fatty chain are essential for inhibitory activity against both *α*-glucosidase and PTP1B. These functional groups stabilize enzyme–ligand complexes through hydrogen-bonding and hydrophobic interactions [20]. Additionally, stereochemistry and substitution patterns further influence biological activity. Yang et al. demonstrated that the substitution and configuration of the hydroxyl group at the C-11 position of the flavanol–fatty alcohol hybrids, as well as the length of the aliphatic chain at the C-13 position, are critical determinants of cytotoxicity toward HepG2 cells. Meanwhile, hybrids with a *β*-configured 3,4-dihydroxyphenyl group exhibited stronger HPL inhibitory activity compared to those with an *α*-configured group [22]. In contrast, another type of flavanol–monoterpene hybrid compound, characterized by a higher content of ring structures, tends to adopt cage-like conformations. This conformation hinders effective interactions with the amino acids within the active site of HPL. The resulting steric hindrance limits productive binding in these flavanol–monoterpene hybrids, thereby reducing or abolishing HPL inhibition [22].

Current studies on flavanol hybrids have primarily focused on their biological activities and underlying structure–activity relationships, whereas their pharmacokinetic properties remain largely unexplored. A recent systematic review of 49 human intervention studies reported a mean bioavailability of 31 ± 23% for flavan-3-ols and identified up to 180 circulating metabolites, highlighting the extensive phase II conjugation and gut microbiota-mediated biotransformation of this class of compounds in vivo [104]. Considering that flavanol hybrids retain the flavanol scaffold while possessing distinct structural modifications, future studies should further elucidate how these structural characteristics affect their pharmacokinetic behavior and biological efficacy.

## 6. Safety and Toxicological Assessment

The safety of ATE has been evaluated in both animal and cellular studies. Park et al. assessed the oral toxicity of ATEE in Balb/c mice over 3 weeks, administering doses of 250, 500, 1000, and 2000 mg/kg/day. The results indicated that no treatment-related adverse effects were observed with respect to mortality, clinical signs, organ function, or body weight. Additionally, hematological and serum biochemical parameters remained within normal ranges and were comparable to those of the control group. These findings indicate that ATEE did not induce observable toxicity in Balb/c mice after 3 weeks of continuous administration of ATEE, with a no-observed-adverse-effect level (NOAEL) greater than 2000 mg/kg/day [105]. Another study conducted an acute oral toxicological assessment of ATE. The results indicated that the maximum tolerated dose exceeded 10 g/kg in both female and male mice, meeting the criteria for classification as a ‘substantially non-toxic substance’ [106]. These results support the safety profile of AT and its potential application in functional foods, cosmetics, and pharmaceuticals.

Furthermore, the cytotoxic properties of AEO and its active components have also attracted attention. Studies have shown that AEO exhibits low toxicity toward normal human cells while exhibiting selective cytotoxicity toward cancer cells [78]. Another study showed that AEO and its major components, eucalyptol and limonene, exhibited notable contact and fumigant toxicity against *Tribolium castaneum* and *Lasioderma serricorne*, suggesting their potential application in the control of grain storage pests [107]. In LPS-induced RAW 264.7 cell experiments, even at concentrations exceeding 300 μM, DDO (**281**) had virtually no effect on cell morphology and viability, supporting its favorable safety profile in cellular models [74]. Additionally, geraniol (**38**), a relatively abundant constituent of AEO, exhibited no obvious local irritation or toxic side effects on the mouse vagina at doses of 0.11, 0.22, and 0.44 g/kg, suggesting a favorable safety profile for the treatment of vulvovaginal candidiasis [108]. However, geraniol (**38**) has also been reported to induce allergic contact dermatitis, indicating that its potential sensitization risk warrants attention [109]. On the other hand, citral, a major constituent of AEO, has been reported to induce oxidative stress and exhibit hepatotoxicity at high doses [110]. Another representative constituent of AEO, 1,8-cineole (**50**), has shown subchronic toxicity in animal studies, with a no-observed-adverse-effect level (NOAEL) of 64.15 mg/kg and a lowest-observed-adverse-effect level (LOAEL) of 192.45 mg/kg. Notably, 1,8-cineole (**50**) affected body weight, hematological parameters, and serum biochemical indices in rats to varying degrees, and induced infectious injury as well as hepatic and renal damage in mice; however, these toxic effects were reversible [111]. Despite these findings, clinical evidence regarding the safety and efficacy of AT in humans remains unavailable. Therefore, well-designed toxicological and clinical studies are needed to establish its safety profile and therapeutic potential.

## 7. Industrial Applications

**Figure 5:**
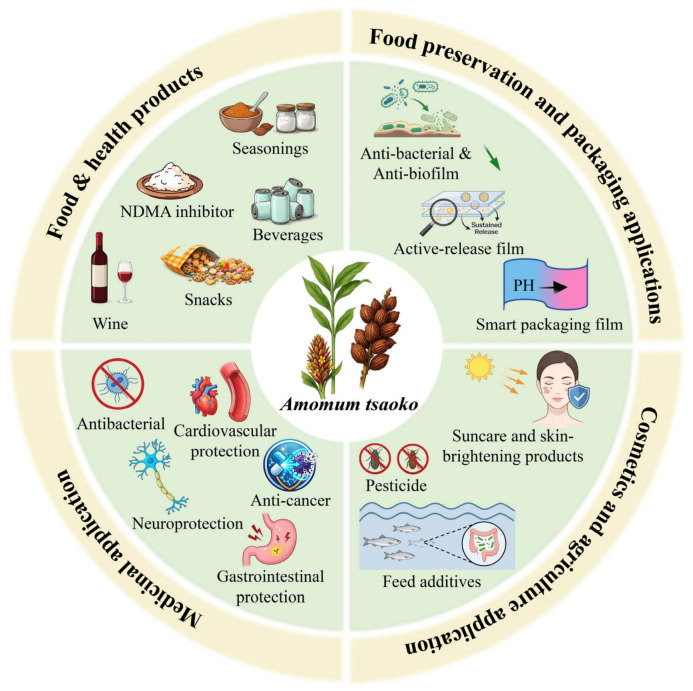
Industrial applications of *Amomum tsaoko* Crevost & Lem. and its by-products.

AT exhibits broad application prospects in the food, pharmaceutical, cosmetics, and agriculture sectors (Figure 5). Its diverse range of applications highlights the adaptability and functional potential of this plant resource across various industrial systems, providing insights for valuable research and development aimed at the sustainable use of natural resources.

### 7.1. Food and Health Products

The dried ripe fruits of AT are widely recognized as a medicinal and edible resource in Southeast Asia and have long been used as spices and flavoring agents [13]. Previous studies have shown that NaNO_2_ may, to some extent, suppress nitrosamine formation by inhibiting the reaction between nitrite and amine compounds, suggesting potential applications in food safety enhancement [112]. In recent years, the development of AT-based products in fermented alcoholic beverages, functional alcoholic drinks, and related foods has largely been reflected in Chinese patent publications. Patented technologies have explored the incorporation of AT into the baijiu daqu system to enhance characteristic aroma profiles, as well as its application in the preparation of specific aromatic baijiu or functional alcoholic beverages (Chinese Patent CN120209950A). Additional developments include AT-based liquid beverages (CN118923783A) and AT-flavored foods (CN116114868A), aimed at broadening its utilization in functional beverages and snack foods while preserving its characteristic flavor. AT has also been proposed for use alone or in combination with other medicinal and edible plants to promote probiotic proliferation, enrich beneficial microbiota, and suppress potentially harmful microorganisms (CN121081578A).

### 7.2. Food Preservation and Packaging Applications

Beyond direct food and health-related products, increasing attention has been directed toward the use of AEO in food preservation and functional packaging systems [113]. Studies have shown that AEO, owing to its antibacterial, anti-biofilm, and antioxidant properties, can function as a natural preservative in diverse food systems, particularly for refrigerated foods, including meat, seafood, fruits, and vegetables. In food preservation applications, AEO has been successfully incorporated into packaging materials through various carrier systems. Microencapsulation and nanoemulsion technologies improve the stability of essential oils and enable controlled release, thereby maintaining sustained antimicrobial activity under low-temperature storage conditions. For example, active films prepared by loading microencapsulated AEO onto a PVA/mixed starch matrix can significantly delay quality deterioration during the storage of seafood and extend shelf life [114]. Similarly, chitosan-based films containing AEO nanoemulsions demonstrate superior overall preservation effects compared to the base material alone in the preservation of fresh meat [115]. In addition to its antibacterial effects, AEO exhibits significant anti-biofilm activity, which is particularly relevant to food safety applications. Relevant studies have demonstrated that AEO can effectively inhibit and disrupt biofilms formed by various foodborne pathogens. The underlying mechanisms involve inhibiting initial adhesion, reducing the secretion of extracellular polymers (EPS), and regulating the expression of biofilm-related genes [116,117]. These properties confer unique advantages on AEO in the preservation of meat products and the control of microorganisms on the surfaces of food contact materials. With advancements in materials science and food engineering, AEO has also been integrated into high-performance active and smart packaging systems. For instance, the stable loading and controlled release of essential oils via nanocarriers can improve the preservation performance of packaging films in the refrigerated preservation of fruits and vegetables [118]. Furthermore, the incorporation of AEO as a natural antimicrobial agent into pH-responsive smart packaging films, in combination with purple potato anthocyanins, enables visual monitoring of the spoilage process in seafood products while simultaneously preserving freshness. This approach broadens the application potential of AEO in the domain of smart food packaging [119].

### 7.3. Medicinal Application

Bioactive compounds in AT have demonstrated significant potential in treating various diseases, including diabetes, cardiovascular diseases, neurological disorders, gastrointestinal diseases, cancer, and inflammatory conditions. These findings support the development of AT-based functional products and natural health formulations.

Traditionally, AT has been widely used as a principal component in herbal formulations. Representative formulations are primarily used for two purposes: the management of gastrointestinal disorders associated with the traditional concept of spleen–stomach cold-damp syndrome and the treatment of infectious diseases, including malaria [120]. Modern research indicates that AT has been shown to improve gastrointestinal function in rats with functional dyspepsia and is clinically used to alleviate symptoms such as abdominal distension and pain [121]. Recent patent evidence has further highlighted the potential application of AEO in oral healthcare products. The results indicate that this essential oil can inhibit the proliferation, biofilm formation, and acid-producing capacity of pathogenic bacteria associated with dental caries, periodontitis, and halitosis, including *Streptococcus mutans*, *Porphyromonas gingivalis*, and *Fusobacterium nucleatum*, supporting the development of AEO-based oral care products in the development of oral care products (CN118909696A).

### 7.4. Cosmetics and Agriculture Applications

Beyond the applications discussed above, AT also shows considerable potential in the cosmetics and agricultural sectors. FAT is primarily characterized by its fatty notes, accompanied by citrus and green nuances, resulting in a well-balanced and intense aroma profile that contributes to its broad use in food and cosmetic products [122]. In the cosmetic field, ATE has been reported to exhibit strong UV-absorbing properties, highlighting its potential application in sunscreen formulations [123]. Patented formulations have further proposed the incorporation of ATE as an active ingredient in emulsion-based products. In addition, ATE has been shown to inhibit tyrosinase activity and reduce melanin production in zebrafish embryos, supporting its application in skin-whitening and skincare products (CN119950392A). In agriculture, AT has been reported to improve the gut microbiota of aquaculture animals, potentially reducing the dependence on conventional antibiotics and highlighting its promise as a sustainable feed additive [90]. Moreover, AEO and its major constituents, eucalyptol and limonene, exhibit insecticidal and fumigant activities against grain storage pests, supporting their potential use as natural insecticides and grain storage protectants [107].

## 8. Conclusions and Future Perspectives

In recent years, AT has attracted significant attention in academic circles because of its dual role as a food and medicinal resource. This review summarizes the bioactive compounds, health functions, industrial application potential, and safety profile of AT. AT is abundant in various bioactive compounds, including flavonoids, diarylheptanoids, phenolic acids, terpenes, steroids, and essential oils. These constituents contribute to its antibacterial, hypoglycemic, antioxidant, anti-inflammatory, anticancer, neuroprotective, anti-obesity, gastroprotective, and immunomodulatory effects. These characteristics highlight its potential for modern therapeutic and industrial applications.

Despite the considerable progress achieved to date, several critical challenges and research gaps remain to be addressed: (a) Current studies on the chemical composition of AT have largely focused on volatile constituents, while comparatively little attention has been devoted to the systematic identification of non-volatile components and their functional contributions. It is necessary to combine metabolomics with functional evaluation methods to systematically analyze and attribute functions to non-volatile components. (b) Existing studies have reported that AT possesses various bioactivities, but the relationship between these activities and their specific medical applications remains poorly understood. Future research should integrate molecular and cellular studies with in vivo experiments to identify key bioactive components and their mechanisms of action, with a particular focus on bioavailability, to deepen our understanding of their potential therapeutic value. (c) Essential oils serve as the primary basis for AT’s distinctive aroma and antimicrobial activity; however, their poor stability, low solubility, and intense flavor may adversely affect food texture and bioactivity. Therefore, it is necessary to develop more efficient essential oil delivery and controlled-release systems to enhance their stability and antimicrobial efficacy. (d) The sensory quality, nutritional composition, and functional properties of AT are strongly influenced by multiple factors, including variety, origin, drying methods, processing techniques, and storage conditions. Future research should focus on establishing standardized processing procedures and a comprehensive quality control system across the entire industrial chain to ensure the quality of AT and promote the healthy development of the industry. (e) Systematic evaluations of AT’s toxicity and mechanisms of action remain relatively insufficient. Therefore, in-depth research in these areas is essential to mitigate the risks associated with product development and clinical applications. (f) Large quantities of stems, leaves, and seeds are often discarded as waste, resulting in resource wastage and environmental pollution. These AT by-products also require commercially viable bioprocessing solutions.

In conclusion, the growing body of evidence highlights AT as a valuable medicinal and edible resource with diverse bioactive constituents, broad health-promoting properties, and promising industrial applications. Addressing the current limitations in phytochemical characterization, mechanistic studies, bioavailability, safety assessment, quality control, and resource utilization will be essential for unlocking its full potential. These efforts will provide a solid scientific foundation for the sustainable development and commercialization of AT in functional foods, nutraceuticals, pharmaceuticals, and other health-related industries.

## Data Availability

No data was used for the research described in the article.

## Supplementary Materials

The following supporting information can be downloaded at: https://www.mdpi.com/article/10.3390/foods15142513/s1, Table S1: Bioactive chemicals isolated from *Amomum tsaoko* Crevost & Lem. References [124,125,126,127,128,129,130] are cited in the Supplementary Materials.

## Glossary

5-HT: 5-hydroxytryptamine	IL-1*β*: interleukin-1*β*
5-HT_2_A: 5-hydroxytryptamine receptor 2A	IL-6: interleukin-6
8-ISO-PGF_2_α: 8-iso-prostaglandin F_2_α	iNOS: inducible nitric oxide synthase
ABTS: 2,2’-azinobis-(3-ethylbenzthiazoline-6-sulphonate)	I*κ*B-*α*: inhibitor of *κ*B alpha
AChE: acetylcholinesterase	JNK: c-Jun n-terminal kinase
AEO: *Amomum tsaoko* essential	LDL: low-density lipoprotein
AKI: acute kidney injury	LPS: lipopolysaccharide
ARE: antioxidant response element	MAPK: mitogen-activated protein kinases
AT: *Amomum tsaoko* Crevost & Lemarié	MBC: minimum bactericidal concentration
ATE: *Amomum tsaoko* extract	MCP-1: monocyte chemotactic protein-1
ATEE: *Amomum tsaoko* ethanol extract	MDA: malondialdehyde
ATF: *Amomum tsaoko* fruits	MIC: minimum inhibitory concentration
ATFME: *Amomum tsaoko* fruit methanol extract	MLC: minimum lethal concentration
ATME: *Amomum tsaoko* methanol extract	MLC3: myosin light chain 3
ATSEE: *Amomum tsaoko* seeds ethanol extract	MLKL: mixed lineage kinase domain-like protein
Bcl-2: B-cell lymphoma 2	NFATc1: nuclear factor of activated T cells, cytoplasmic 1
BDNF: brain-derived neurotrophic factor	NF-*κ*B: nuclear factor-*κ*B
BF: n-butanol fraction	NLRP3: NOD-like receptor protein 3
Ca2+/CaMKII: calcium/calmodulin-dependent protein kinase II	NO: nitric oxide
CC50: half maximal cytotoxic concentration	Nrf2: nuclear factor erythroid 2-related factor 2
CG-A: 2,3-dihydro-2-(4′-hydroxyphenylethyl)-6-[(3″,4″-dihydroxy-5″-methoxy) phenyl]-4-pyrone	*p*-ERK: phosphorylated extracellular signal-regulated kinase
CG-B: 4-dihydro-2-(4′-hydroxyphenylmethyl)-6-[(3″,4″-dihydroxy-5″-methoxyphenyl) methylene]-pyran-3,5-dione	PKA: protein kinase A
COX-2: cyclooxygenase-2	PLA_2_: phospholipase A_2_
CREB: cAMP-response element binding protein	*p*-p65: phosphorylated-p65
CYP7A1: cholesterol 7α-hydroxylase	*p*-STAT3: phosphorylated signal transducer and activator of transcription 3
DAT: died *Amomum tsaoko*	PTP1B: protein tyrosine phosphatase 1B
DPPH: 2,2-diphenyl-1-pyridyl hydrazine radical	RAW 264.7: mouse monocyte macrophage leukemia cell line
EF: ethyl acetate fraction	RBD: receptor-binding domain
ERK: extracellular signal-regulated kinase	RIPK1: receptor-interacting protein kinase 1
FAT: Fresh *Amomum tsaoko*	RIPK3: receptor-interacting protein kinase 3
Fos: FBJ murine osteosarcoma viral oncogene homolog	ROS: oxygen species
FRAP: ferric reducing antioxidant power	SARS-CoV-2: severe acute respiratory syndrome coronavirus 2
GABA: gamma-aminobutyric acid	SCFAs: short-chain fatty acids
GLP-1: glucagon-like peptide-1	SOD: superoxide dismutase
GPa: glycophorin A	SPHK1: sphingosine kinase 1
GSH: glutathione	SPHK2: sphingosine kinase 2
GSH-Px: glutathione peroxidase	T2DM: type 2 diabetes
H_2_O_2_: hydrogen peroxide	TBARS: thiobarbituric acid reactive substances
hACE2: human angiotensin-converting enzyme 2	TC: total cholesterol
HMGB1: high mobility group box 1	TLR4: toll-like receptor 4
HO-1: heme oxygenase-1	TNF-α: tumor necrosis factor-α
HPL: human pancreatic lipase	TrkB: tropomyosin receptor kinase B
IC_50_: half maximal inhibitory concentration	TRPA1: transient receptor potential ankyrin 1
IL-10: interleukin-10	VEGF: vascular endothelial growth factor
