# Supplementary material for: Unveiling the Value of Amomum tsaoko Crevost & Lem.: A Review from Bioactive Compounds to Health Benefits and Industrial Applications
Source: Foods. 2026 Jul 16;15(14):2513. doi: 10.3390/foods15142513 (PMC13409399; doi:10.3390/foods15142513)
Supplement: Supplementary file 1 [file foods-15-02513-s001.zip › foods-4405266-supplementary.pdf]

Table S1 Bioactive chemicals isolated from *Amomum tsaoko* Crevost & Lem.

No.	Classify	chemical compositions	CAS	Molecular formula	Plant parts	Reference
	Aromatic active components					
1		*α*-pinene	80-56-8	$C_{10}H_{16}$	Cobs, FAT, DAT	[10, 16]
2		*β*-myrcene	123-35-3	$C_{10}H_{16}$	Cobs, DAT	[10, 16]
3		Acetoin	513-86-0	$C_4H_8O_2$	Cobs	[16]
4		4-thujanol	208-911-7	$C_{10}H_{18}O$	Cobs	[16]
5		4-heptyn-3-ol	32398-69-9	$C_7H_{12}O$	Cobs	[16]
6		Nonanal	124-19-6	$C_9H_{18}O$	Cobs, DAT	[10, 16]
7		2-3-methylfuran		C_6H_8O	Cobs	[16]
8		5-methyl-2-heptanol	54630-50-1	$C_8H_{18}O$	Cobs	[16]
9		6-methyl-5-hepten-2-ol	1569-60-4	$C_8H_{16}O$	Cobs	[16]
10		Theaspirane	36431-72-8	$C_{13}H_{22}O$	Cobs	[16]
11		Thujopsene	470-40-6	$C_{15}H_{24}$	Cobs	[16]
12		(*E*)-2-nonenal	18829-56-6	$C_9H_{16}O$	Cobs, DAT	[10, 16]
13		Caryophyllene	87-44-5	$C_{15}H_{24}$	Cobs	[16]
14		(*Z*)-2-decenal	2497-25-8	$C_{10}H_{18}O$	Cobs, FAT,	[10, 16]

					DAT	
15		Neral	106-26-3	$C_{10}H_{16}O$	Cobs, FAT, DAT	[10, 16]
16		*β*-dihydroagarofuran	5956-09-2	$C_{15}H_{26}O$	Cobs	[16]
17		*α*-terpineol	98-55-5	$C_{10}H_{18}O$	Cobs, FAT, DAT	[10, 16]
18		*γ*-muurolene	30021-74-0	$C_{15}H_{24}$	Cobs	[16]
19		Geranial	141-27-5	$C_{10}H_{16}O$	Cobs, FAT, DAT	[10, 16]
20		Cubenene	29837-12-5	$C_{15}H_{24}$	Cobs	[16]
21		2-decen-1-ol	22104-80-9	$C_{10}H_{20}O$	Cobs	[16]
22		Calamenene	483-77-2	$C_{15}H_{22}$	Cobs	[16]
23		Geranyl isovalerate	109-20-6	$C_{15}H_{26}O_2$	Cobs	[16]
24		Cubebol	23445-02-5	$C_{15}H_{26}O$	Cobs	[16]
25		(*E*)-sesquisabinene hydrate	119238-96-9	$C_{15}H_{26}O$	Cobs	[16]
26		Ledol	577-27-5	$C_{15}H_{26}O$	Cobs	[16]
27		Epicubenol	19912-67-5	$C_{15}H_{26}O$	Cobs	[16]
28		T-muurolol	19912-62-0	$C_{15}H_{26}O$	Cobs	[16]
29		*α*-eudesmol	473-16-5	$C_{15}H_{26}O$	Cobs	[16]
30		Farnesal	19317-11-4	$C_{15}H_{24}O$	Cobs	[16]
31		Farnesol	4602-84-0	$C_{15}H_{26}O$	Cobs	[16]
32		(*E*)-2-dodecenal	20407-84-5	$C_{12}H_{22}O$	FAT,	[14]

					DAT	
33		(E)-2-octenal	2548-87-0	$C_8H_{14}O$	FAT, DAT	[14]
34		Octanal	124-13-0	$C_8H_{16}O$	FAT, DAT	[14]
35		Eucalyptol	470-82-6	$C_{10}H_{18}O$	FAT, DAT	[14]
36		(E)-2-decenal	3913-81-3	$C_{10}H_{18}O$	FAT, DAT	[14]
37		Linalool	78-70-6	$C_{10}H_{18}O$	FAT, DAT	[14]
38		Geraniol	106-24-1	$C_{10}H_{18}O$	FAT, DAT	[10, 14]
39		α-Phellandrene	99-83-2	$C_{10}H_{16}$	FAT, DAT	[14]
40		β-Pinene	127-91-3	$C_{10}H_{16}$	FAT, DAT	[14]
41		Sabinen	3387-41-5	$C_{10}H_{16}$	FAT, DAT	[14]
42		*Trans*-nerolidol	7212-44-4	$C_{15}H_{26}O$	FAT, DAT, seeds	[3, 4]
43		D-limonene	5989-27-5	$C_{10}H_{16}$	FAT, DAT	[10, 14]
44		Decanal	112-31-2	$C_{10}H_{20}O$	FAT	[14]
45		Ocimene	29714-87-2	$C_{10}H_{16}$	FAT,	[14]

					DAT	
46		Elemol	639-99-6	$C_{15}H_{26}O$	FAT, DAT	[14]
47		(-)-Terpinen-4-ol	20126-76-5	$C_{10}H_{18}O$	FAT, DAT	[14]
48		2-Nonanone	821-55-6	$C_9H_{18}O$	FAT, DAT	[10, 14]
49		L-*α*-Terpineol	10482-56-1	$C_{10}H_{18}O$	FAT, DAT	[14]
50		1,8-cineole	470-82-6	$C_{10}H_{18}O$	DAT	[10]
51		Hexanal	66-25-1	$C_6H_{12}O$	DAT	[10]
52		(*E*, *E*)-2,4-dodecadienal	21662-16-8	$C_{12}H_{20}O$	DAT	[10]
53		*E*-nerolidol	40716-66-3	$C_{15}H_{26}O$	DAT	[10]
54		Sabinene	3387-41-5	$C_{10}H_{16}$	DAT	[10]
55		6-methyl-5-hepten-2-one	110-93-0	$C_8H_{14}O$	DAT	[10]
56		4-terpeneol	562-74-3	$C_{10}H_{18}O$	DAT	[10]
57		Citronellal	106-23-0	$C_{10}H_{18}O$	DAT	[10]
58		2-nonanol	628-99-9	$C_9H_{20}O$	DAT	[10]
	Flavonoids					
59		Alpinetin	1090-65-9	$C_{16}H_{14}O_4$	Fruits	[124]
60		Naringenin-5-O-methyl ether		$C_{16}H_{14}O_5$	Fruits	[124]
61		Naringenin	480-41-1	$C_{15}H_{12}O_5$	Fruits	[124]
62		Hesperetin	520-33-2	$C_{16}H_{14}O_6$	Fruits	[124]
63		2',4',6'- trihydroxy-4-methoxy chalcone	62014-87-3	$C_{16}H_{14}O_5$	Fruits	[124]
64		Boesenbergin B	93208-46-9	$C_{26}H_{28}O_4$	Fruits	[124]

65		4-hydroxyboesenbergin B		$C_{26}H_{28}O_5$	Fruits	[124]
66		(-)-epi-afzelechin	24808-04-6	$C_{15}H_{14}O_5$	Fruits	[19]
67		(+)-afzelechin	2545-00-8	$C_{15}H_{14}O_5$	Fruits	[19]
68		(+)-epicatechin	35323-91-2	$C_{15}H_{14}O_6$		[75]
69		(-)-catechin	18829-70-4	$C_{15}H_{14}O_6$	Fruits	[95]
70		8-aldehyde-catechin		$C_{16}H_{14}O_7$	Fruits	[19]
71		Sappanone B	104778-15-6	$C_{16}H_{14}O_6$	Fruits	[19]
72		Brazilin	474-07-7	$C_{16}H_{14}O_5$	Fruits	[19]
73		Quercetin	117-39-5	$C_{15}H_{10}O_7$	Fruits	[17]
74		Quercetin-7-O-*β*-glucoside	482-35-9	$C_{21}H_{20}O_{12}$	Fruits	[17]
75		Quercetin-3-O-*β*-glucoside		$C_{21}H_{20}O_{12}$	Fruits	[17]
76		Rutin	153-18-4	$C_{27}H_{30}O_{16}$		[125]
77		(2*R*,3*R*,4*R*)-3',5'-dimethoxy-3,4,7,4'-tetrahydroxy-flavan		$C_{17}H_{18}O_7$	Fruits	[95]
78		4'-hydroxy-2'-methoxychalcone	69470-84-4	$C_{16}H_{14}O_3$	Fruits	[95]
79		4,4'-dimethoxychalcone	2373-89-9	$C_{17}H_{16}O_3$	Fruits	[95]
80		4',7-dihydroxy-3',6-diprenylflavone	91433-17-9	$C_{25}H_{26}O_4$	Fruits	[95]
81		3',7-dihydroxy-4'-methoxy-flavan	76426-35-2	$C_{16}H_{16}O_4$	Fruits	[95]
82		2',4'-dihydroxy-4-methoxychalcone	13351-10-5	$C_{16}H_{14}O_4$	Fruits	[95]
83		2',4,4'-trimethoxychalcone	18493-34-0	$C_{18}H_{18}O_4$	Fruits	[95]
84		Abyssinoflavanone VII	943515-83-1	$C_{25}H_{28}O_6$	Fruits	[95]
85		4-hydroxy-4'-methoxychalcone	69704-15-0	$C_{16}H_{14}O_3$	Fruits	[95]
86		Quercetin-3-O-*β*-glucopyranoside	21637-25-2	$C_{21}H_{20}O_{12}$	Fruits	[125]
87		3',5'-di-C-*β*-D-glucopyranosylphloretin		$C_{21}H_{26}O_9$	Fruits	[125]
88		Tsaokonol A		$C_{26}H_{28}O_5$	Fruits	[126]
89		Tsaokonol B		$C_{26}H_{28}O_5$	Fruits	[126]

90		Tsaokonol C		$C_{26}H_{28}O_4$	Fruits	[126]
91		Tsaokonol D		$C_{26}H_{28}O_4$	Fruits	[126]
92		Tsaokonol E		$C_{31}H_{36}O_5$	Fruits	[126]
93		Tsaokonol F		$C_{31}H_{36}O_5$	Fruits	[126]
94		Tsaokonol G		$C_{31}H_{36}O_4$	Fruits	[126]
95		Tsaokonol H		$C_{31}H_{36}O_4$	Fruits	[126]
96		Tsaokonol I		$C_{31}H_{36}O_5$	Fruits	[126]
97		Tsaokonol J		$C_{31}H_{36}O_4$	Fruits	[126]
98		2-(4-hydroxy-3-methoxybenzoyl)-4-methoxy-benzaldehyde		$C_{15}H_{14}O_5$	Fruits	[95]
99		Epicatechin-(4*β*→8,2*β*→O→7)-epi-afzelechin		$C_{30}H_{24}O_{11}$	Fruits	[19]
100		Proanthocyanidin A-2	41743-41-3	$C_{30}H_{24}O_{12}$	Fruits	[19]
101		Amomutsaokin A		$C_{25}H_{28}O_6$	Fruits	[19]
102		Amomutsaokin B		$C_{25}H_{28}O_6$	Fruits	[19]
103		Amomutsaokin C		$C_{25}H_{28}O_6$	Fruits	[19]
104		Amomutsaokin D		$C_{25}H_{28}O_6$	Fruits	[19]
105		Amomutsaokin E		$C_{25}H_{30}O_7$	Fruits	[19]
106		Amomutsaokin F		$C_{25}H_{30}O_7$	Fruits	[19]
107		Amomutsaokin G		$C_{25}H_{30}O_7$	Fruits	[19]
108		Amomutsaokin H		$C_{25}H_{30}O_7$	Fruits	[19]
109		Flavanocoumarin		$C_{18}H_{14}O_7$	Fruits	[19]
110		Tsaokoflavanol A		$C_{23}H_{28}O_7$	Fruits	[20]
111		Tsaokoflavanol B		$C_{25}H_{32}O_7$	Fruits	[20]
112		Tsaokoflavanol C		$C_{25}H_{32}O_7$	Fruits	[20]
113		Tsaokoflavanol D		$C_{25}H_{30}O_8$	Fruits	[20]
114		Tsaokoflavanol E		$C_{27}H_{32}O_9$	Fruits	[20]

115		Tsaokoflavanol F		$C_{27}H_{36}O_7$	Fruits	[20]
116		Tsaokoflavanol G		$C_{23}H_{28}O_7$	Fruits	[20]
117		Tsaokoflavanol H		$C_{23}H_{28}O_7$	Fruits	[20]
118		Tsaokoflavanol I		$C_{23}H_{28}O_7$	Fruits	[20]
119		Tsaokoflavanol J		$C_{25}H_{32}O_7$	Fruits	[20]
120		Tsaokoflavanol K		$C_{25}H_{32}O_7$	Fruits	[20]
121		Tsaokoflavanol L		$C_{25}H_{32}O_7$	Fruits	[20]
122		Tsaokoflavanol M		$C_{25}H_{32}O_7$	Fruits	[20]
123		Tsaokoflavanol N		$C_{25}H_{30}O_8$	Fruits	[20]
124		Tsaokoflavanol O		$C_{25}H_{30}O_8$	Fruits	[20]
125		Tsaokoflavanol P		$C_{27}H_{32}O_9$	Fruits	[20]
126		Tsaokoflavanol Q		$C_{27}H_{32}O_9$	Fruits	[20]
127		Tsaokoflavanol R		$C_{27}H_{36}O_7$	Fruits	[20]
128		Tsaokoflavanol S		$C_{27}H_{36}O_7$	Fruits	[20]
129		Tsaokoflavanol A1		$C_{24}H_{30}O_7$	Fruits	[22]
130		Tsaokoflavanol B1		$C_{24}H_{31}O_7$	Fruits	[22]
131		Tsaokoflavanol C1		$C_{26}H_{34}O_7$	Fruits	[22]
132		Tsaokoflavanol D1		$C_{26}H_{34}O_7$	Fruits	[22]
133		Tsaokoflavanol E1		$C_{28}H_{38}O_7$	Fruits	[22]
134		Tsaokoflavanol F1		$C_{23}H_{28}O_7$	Fruits	[22]
135		Tsaokoflavanol G1		$C_{24}H_{31}O_7$	Fruits	[22]
136		Tsaokoflavanol H1		$C_{24}H_{31}O_7$	Fruits	[22]
137		Tsaokoflavanol I1		$C_{26}H_{34}O_6$	Fruits	[22]
138		Tsaokoflavanol J1		$C_{25}H_{27}O_7$	Fruits	[22]
139		Tsaokol B		$C_{25}H_{26}O_7$	Fruits	[21]

140		Tsaokol A		$C_{25}H_{26}O_7$	Seeds, fruits	[21, 28]
	Diarylheptanoids					
141		2,3-dihydro-2- (4′-hydroxy-phenylethyl)-6-[(3″,4″-dihydroxy-5″-methoxy) phenyl]-4-pyrone		$C_{20}H_{20}O_6$	Fruits	[64]
142		4-dihydro-2-(4′-hydroxy-phenylmethyl)-6-[(3″,4″-dihydroxy-5″-methoxyphenyl) methylene]-pyran-3,5-dione		$C_{20}H_{18}O_7$	Fruits	[64]
143		6-(4-Hydroxyphenyl)-4-hydroxyhexan-2-one		$C_{12}H_{16}O_3$	Fruits	[127]
144		tsaokoarylone/7-(4-hydroxy-3-methoxyphenyl)-1-(4-hydroxyphenyl)-hepta-4E,6E-dien-3-one	811471-20-2	$C_{20}H_{20}O_4$	Fruits	[127]
145		1,7-bis (4-hydroxyphenyl) hepta-4E,6E-dien-3-one	332371-82-1	$C_{19}H_{18}O_3$	Fruits	[127]
146		Amomutsaokol A		$C_{31}H_{34}O_{11}$	Fruit	[56]
147		Amomutsaokol B		$C_{29}H_{32}O_6$	Fruit	[56]
148		Amomutsaokol C		$C_{20}H_{22}O_6$	Fruit	[56]
149		Amomutsaokol D		$C_{20}H_{18}O_6$	Fruit	[56]
150		Amomutsaokol E		$C_{20}H_{20}O_6$	Fruit	[56]
151		Amomutsaokol F		$C_{22}H_{26}O_8$	Fruit	[56]
152		Amomutsaokol G		$C_{19}H_{22}O_4$	Fruit	[56]
153		Amomutsaokol H		$C_{20}H_{24}O_5$	Fruit	[56]
154		Amomutsaokol I		$C_{22}H_{26}O_5$	Fruit	[56]
155		Amomutsaokol J		$C_{20}H_{20}O_4$	Fruit	[56]
156		Amomutsaokol K		$C_{20}H_{24}O_5$	Fruit	[56]
157		(3*R*,5*R*)-3,5-dihydroxy-1,7-bis (4-hydroxyphenyl) heptane		$C_{19}H_{24}O_4$	Fruit	[56]
158		(3*R*,5*R*)-3-acetoxy-5- hydroxy-1,7-bis(4-hydroxyphenyl) heptane		$C_{21}H_{26}O_5$	Fruit	[56]
159		(3*R*,5*R*)-3,5-dihydroxy-1-(3,4-dihydroxyphenyl)-7-(4-hydroxyphenyl) heptane	408324-00-5	$C_{19}H_{24}O_5$	Fruit	[56]

160		(3*R*,5*R*)-3,5-dihydroxy-1-(4-hydroxy-3-methoxyphenyl)-7-(4-hydroxyphenyl) heptane		$C_{20}H_{26}O_5$	Fruit	[56]
161		Meso-hannokinol	79055-11-1	$C_{19}H_{24}O_4$	Leaves , fruit	[56-57]
162		Rel-(3*R*,5*S*)-3,5-dihydroxy-1-(3,4-dihydroxyphenyl)-7-(4-hydroxyphenyl) heptane		$C_{19}H_{24}O_5$	Fruit	[56]
163		(3*R*,5*S*)-3,5-dihydroxy-1-(4-hydroxy-3-methoxyphenyl)-7-(4-hydroxy phenyl) heptane		$C_{20}H_{26}O_5$	Fruit	[56]
164		Rel-(3*R*,5*S*)-3,5-dihydroxy-1-(4-hydroxy-3-methoxy phenyl)-7-(3,4-dihydroxyphenyl) heptane		$C_{21}H_{26}O_5$	Fruit	[56]
165		4-[(3*S*,5*E*)-3-hydroxy-7-(4-hydroxyphenyl) hept-5-en-1-yl]-2-methoxyphenol		$C_{20}H_{24}O_4$	Fruit	[56]
166		1,7-bis(4-hydroxyphenyl)-3-hepten-5-one		$C_{19}H_{20}O_3$	Fruit	[56]
167		(4*E*,6*E*)-1,7-bis (4-hydroxyphenyl) hepta-4,6-dien-3-one	332371-82-1	$C_{19}H_{18}O_3$	Fruit	[56]
168		(4*E*,6*E*)-1,7-bis(4-hydroxy-3-methoxyphenyl) hepta-4,6-dien-3-one		$C_{21}H_{22}O_5$	Fruit	[56]
169		2-hydroxymusaitinerin A		$C_{41}H_{44}O_{13}$	Leaves	[57]
170		5''-methoxymusaitinerin A		$C_{42}H_{46}O_{13}$	Leaves	[57]
171		Amomumanoid A		$C_{21}H_{24}O_6$	Leaves	[57]
172		Amomumanoid B		$C_{21}H_{24}O_6$	Leaves	[57]
173		(3*S*,5*S*,6*R*,7*R*)-5,6-dihydroxy-1,7-bis(4-hydroxyphenyl)-de-O-methylcentrolobine		$C_{19}H_{22}O_5$	Leaves	[57]
174		(+)-hannokinol	408324-76-5	$C_{19}H_{24}O_4$	Leaves , fruit	[38, 57]
175		Kravanhol F			Leaves	[57]
176		1,7-bis[4-hydroxyphenyl]-3-hepten-5-one		$C_{19}H_{20}O_3$	Leaves	[57]
177		(1*E*,4*E*)-1,7-bis (4-hydroxy-3-methoxyphenyl) hepta-1,4-dien-3-one		$C_{21}H_{22}O_5$	Leaves	[57]
178		(2*S*,4*S*,6*R*)-2-((S)-1-hydroxy-2-(4-hydroxy-3-methoxyphenyl) ethyl)-6- (4-hydroxy-3,5-dimethoxyphenyl) tetrahydro-2H-pyran-4-ol		$C_{22}H_{28}O_8$	Leaves	[57]
179		Platyphyllone		$C_{19}H_{22}O_4$	Leaves	[57]
180		Musaitinerin A		$C_{41}H_{44}O_{14}$	Leaves	[57]

181		1,7-bis (4-hydroxy-3-methoxyphenyl)-4,6-heptadien-3-one		$C_{21}H_{22}O_5$	Fruits	[66]
182		Hannokinin /4,4′-dihydroxyashabushiketol		$C_{19}H_{22}O_4$	Fruits	[66]
183		1,7-bis(4-hydroxyphenyl)-4(*E*)-hepten-3-one		$C_{19}H_{20}O_3$	Fruits	[95]
184		Tsaokopyranol A		$C_{28}H_{32}O_9$	Fruits	[53]
185		Tsaokopyranol B		$C_{29}H_{34}O_{10}$	Fruits	[53]
186		Tsaokopyranol C		$C_{21}H_{26}O_8$	Fruits	[53]
187		Tsaokopyranol D		$C_{22}H_{28}O_9$	Fruits	[53]
188		Tsaokopyranol E		$C_{20}H_{24}O_6$	Fruits	[53]
189		Tsaokopyranol F		$C_{20}H_{24}O_6$	Fruits	[53]
190		Tsaokopyranol G		$C_{21}H_{26}O_8$	Fruits	[53]
191		Tsaokopyranol H		$C_{20}H_{24}O_5$	Fruits	[53]
192		Tsaokopyranol I		$C_{20}H_{24}O_6$	Fruits	[53]
193		Tsaokopyranol J		$C_{21}H_{24}O_7$	Fruits	[53]
194		Tsaokopyranol K		$C_{20}H_{24}O_6$	Fruits	[53]
195		Tsaokopyranol L		$C_{20}H_{22}O_4$	Fruits	[53]
196		Tsaokopyranol M		$C_{20}H_{22}O_4$	Fruits	[53]
197		(2*R*,6*S*)-3,4-dehydro-1,7-bis (4-hydroxy phenyl)-4′-de-O-methyl centrolobine		$C_{19}H_{22}O_3$	Fruits	[53]
198		(2*R*,6*R*)-3,4-dehydro-4′-de-O-methyl centrolobin		$C_{19}H_{22}O_3$	Fruits	[53]
199		Phaeoheptanoxide		$C_{19}H_{22}O_5$	Fruits	[53]
200		Engelheptanoxides C		$C_{20}H_{24}O_5$	Fruits	[53]
201		4-(1-hydroxypropyl) phenol-ethyl-4-hydroxy-(*S*)-benzenemethanol		$C_{16}H_{18}O_4$	Fruits	[95]
	Phenolic acids					
202		Pyrogallic acid	87-66-1	$C_6H_6O_3$	Fruits	[125]
203		3,4-dimethoxybenzoic acid	93-07-2	$C_9H_{10}O_4$	Seeds	[28]
204		3-O-methylgallic acid	3934-84-7	$C_8H_8O_5$	Fruits	[38]

205		*p*-hydroxybenzoic acid	99-96-7	$C_7H_6O_3$	Fruits	[38]
206		3,4-dihydroxybenzoic acid	99-50-3	$C_7H_6O_4$	Fruits	[38]
207		(*E*)-pcoumaric acid	501-98-4	$C_9H_8O_3$	Fruits	[38]
208		Protocatechualdehyde	139-85-5	$C_7H_6O_3$	Fruits	[128]
209		vanillic acid	121-34-6	$C_8H_8O_4$	Fruits	[38]
210		Protocatechuic acid	99-50-3	$C_7H_6O_4$	Fruits	[128]
211		3-hydroxybenzoic acid	99-06-9	$C_8H_8O_3$	Fruits	[95]
212		4-hydroxy-benzaldehyde	123-08-0	$C_7H_6O_2$	Seeds	[28]
213		4-methoxy-3-hydroxy-benzaldehyde	621-59-0	$C_8H_8O_3$	Seeds	[28]
214		Vanillin / 4-hydroxy-3-methoxy-benzaldehyde	121-33-5	$C_8H_8O_3$	Seeds, fruits	[28-29]
215		4-hydroxy-2,5- dimethoxy-benzaldehyde	80749-72-0	$C_9H_{10}O_4$	Fruits	[95]
216		4-methoxybenzaldehyde	123-11-5	$C_8H_8O_2$	Fruits	[95]
217		2-methoxy-benzaldehyde	135-02-4	$C_8H_8O_2$	Fruits	[95]
218		3-methoxy-benzaldehyde	591-31-1	$C_8H_8O_2$	Fruits	[95]
219		3-hydroxy-4-methoxybenzaldehyde	621-59-0	$C_8H_8O_4$	Fruits	[95]
220		3-methoxy-4-hydroxy-benzaldehyde	121-33-5	$C_8H_8O_3$	Fruits	[95]
221		2,6-dimethoxy-4-[(1*R*)-1-methoxyethyl]-phenol		$C_{11}H_{16}O_4$	Seeds	[28]
222		2,6-dimethoxy-4-[(1*R*)-1-methoxypropyl]-phenol		$C_{12}H_{18}O_4$	Seeds	[28]
223		2,6-dimethoxy-phenol	91-10-1	$C_8H_{10}O_3$	Seeds	[28]
224		2,6-dimethoxy-4-methyl-phenol	6638-05-7	$C_9H_{12}O_3$	Seeds	[28]
225		2,6-dimethoxy-4-(methoxymethyl)-phenol		$C_{10}H_{14}O_4$	Seeds	[28]
226		1,3-dimethoxybenzene	151-10-0	$C_8H_{10}O_2$	Fruits	[95]
227		1-(4-hydroxy-3,5-dimethoxyphenyl)-1-propanone	5650-43-1	$C_{11}H_{14}O_4$	Seeds	[28]
228		1-(4-hydroxy-3-methoxyphenyl)-ethanone	498-02-2	$C_9H_{10}O_3$	Seeds	[28]

229		Phloroacetophenone 2′-O-glucoside		$C_{14}H_{18}O_9$	Fruits	[89]
230		4-(2-hydroxypropyl) phenol	22805-43-2	$C_9H_{12}O_2$	Fruits	[95]
231		2-methoxy-hydroquinone	824-46-4	$C_7H_8O_3$	Fruits	[95]
232		Hydroquinone	123-31-9	$C_6H_6O_2$	Fruits	[95]
233		Catechol	120-80-9	$C_6H_6O_2$	Fruits	[17]
234		4-methoxy-catechol	3934-97-2	$C_7H_8O_3$	Fruits	[95]
235		2,6-dimethoxy-4-(2-propen-1-yl)-phenol	6627-88-9	$C_{10}H_{14}O_4$	Seeds	[28]
236		1-(4-hydroxy-3-methoxyphenyl)-1-propanone	1835-14-9	$C_{10}H_{12}O_3$	Seeds	[28]
237		Anisole	100-66-3	C_7H_8O	Fruits	[95]
238		3-methoxy-catechol	934-00-9	$C_7H_8O_3$	Fruits	[95]
239		2-methoxyresorcinol	29267-67-2	$C_7H_8O_3$	Fruits	[95]
240		3,3',5,5'-tetramethoxy-[1,1'-biphenyl]-4,4'-diol	612-69-1	$C_{16}H_{18}O_6$	Seeds	[28]
241		*p*-hydroxybenzene propanoic acid	501-97-3	$C_9H_{10}O_3$		[129]
242		2,6-dimethoxyphenol	91-10-1	$C_8H_{10}O_3$	Fruits	[128]
	Terpenes					
243		Amotsaokonal B		$C_{20}H_{32}O$	Fruits	[72]
244		Amotsaokonal C		$C_{20}H_{32}O$	Fruits	[72]
245		(1*RS,*5*SR*,6*RS*)-5-hydroxybicyclo [4.3.0] non-2-ene-2-carbaldehyde		$C_{10}H_{16}O_2$	Fruits	[80]
246		8-hydroxy-2,6-dimethyl-1,6-octadien-3-one		$C_{10}H_{16}O_2$	Seeds	[28]
247		(2*E*,6*E*)-8-(acetyloxy)-2,6-dimethyl-2,6-octadienal		$C_{12}H_{18}O_3$	Seeds	[28]
248		(3*E*)-4- [(1*S*,4a*S*,8a*S*)-decahydro-5,5,8a-trimethyl-2-methylene-1- naphthalenyl]-3-buten-2-one		$C_{18}H_{28}O$	Seeds	[28]
249		8-oxogeraniol	7659-86-1	$C_{10}H_{16}O_2$	Fruits	[66]
250		*p*-menth-1-ene-5,6-diol	3407-42-9	$C_{10}H_{18}O_2$	Fruits	[66]
251		3*α*-hydroxycarvotagenone		$C_{10}H_{16}O_2$	Fruits	[66]

252		(3*S*,4*S*,6*R*)-3,6-dihydroxy-1-menthene		$C_{10}H_{18}O_2$	Fruits	[76]
253		(1*R*,3*S*,4*R*)-3-hydroxy isopulegol		$C_{10}H_{18}O_2$	Fruits	[76]
254		(1*R*,4*S*,6*S*)-1,6-dihydroxy-2-menthene		$C_{10}H_{13}O_2$	Fruits	[76]
255		3,7-dimethyl-2-octene-1,8-diol	18479-58-8	$C_{10}H_{20}O_2$	Fruits	[76]
256		(+)-*trans*-2,3,3a,7a-tetrahydro-1H-indene-4-carbaldehyde	76554-12-4	$C_{10}H_{12}O_4$		[130]
257		Kravanhin C		$C_{20}H_{27}O_4$	Leaves	[33]
258		3-epi-kravanhin A		$C_{20}H_{28}O_4$	Leaves	[33]
259		Kravanhin A		$C_{20}H_{28}O_4$	Leaves	[33]
260		6,7-dihydroxyindan-4-carbaldehyde		$C_{10}H_{10}O_3$	Fruits	[66]
261		6-hydroxyindan-4-carbaldehyde		$C_{10}H_{10}O_2$	Fruits	[66, 95]
262		Isotsaokoin	695188-24-0	$C_{10}H_{14}O_2$	Fruits	[64]
263		Tsaokoin	343605-41-4	$C_{10}H_{14}O_2$	Fruits	[80]
264		Tsaokoic acid / rel-(1*R*,5*R*,6*S*)-5-hydroxybicyclo [4,3,0] non-2-ene-2-carboxaldehyde		$C_{10}H_{14}O_3$	Fruits	[29]
265		6,7-dihydroxy-4-indancarbaldehyde		$C_{10}H_{10}O_3$	Fruits	[95]
266		Amotsaokonal A		$C_{20}H_{30}O$	Fruits	[72]
267		(2*E*,6*E*)-8-hydroxy-2,6-dimethyl-2,6-octadienal	38290-51-6	$C_{10}H_{16}O_2$	Fruits	[80]
268		(2*E*,6*E*)-8-hydroxy-2,6-dimethyl-2,6-octadienal acetate		$C_{12}H_{18}O_3$	Fruits	[80]
269		Coronadiene	17078-12-7	$C_{17}H_{26}O_2$	Fruits	[38]
270		5-indancarbaldehyde	2131-54-0	$C_{10}H_{10}O$	Fruits	[95]
271		4-indancarbaldehyde	57378-68-4	$C_{10}H_{10}O$	Fruits	[95]
272		*R*-5-(1*R*,5*S*-dimethyl-3*R*,4*R*,8*S*-trihydroxy-7-oxabicyclo[3,2,1]-oct-8-yl)-3-methyl-2*Z*,4*E*-pentadienoic acid		$C_{15}H_{20}O_6$		[129]
273		(+)-loliolide	5989-02-6	$C_{11}H_{16}O_3$		[129]
	Steroids					
274		*β*-sitosterol	83-46-5	$C_{29}H_{50}O$	Fruits	[128]

275		*β*-sitosterol 3-O-glucoside/daucosterol	474-58-8	$C_{35}H_{60}O_6$	Fruits	[17, 128]
	Others					
276		(2*E*,8*E*)-10-hydroxy-decadienal	557-48-2	$C_{10}H_{16}O_2$	Fruits	[80]
277		(2*E*)-decenol	18409-17-1	$C_{10}H_{20}O$	Fruits	[80]
278		Acetoxytsaokol A		$C_{14}H_{22}O_4$	Seeds	[28]
279		(2*E*)-Dodecenyl acetate		$C_{14}H_{26}O_2$	Fruits	[72]
280		(2*E*)-1-acetate 2-dodecen-1-ol		$C_{14}H_{26}O_3$	Seeds	[28]
281		(2*E*,8*E*)-2,8-decadiene-1,10-diol		$C_{10}H_{18}O_2$	Seeds	[28]
282		6,7-dihydroxy-3,7- dimethyl-oct-2-enoic acid		$C_{10}H_{18}O_4$	Fruits	[66]
283		Methyl linolenate	301-00-8	$C_{19}H_{32}O_2$	Fruits	[89]
284		(9*S*,10*E*,12*Z*)-9- hydroxy-10,12-octadecadienoic acid	10075-07-7	$C_{10}H_{18}O_4$	Seeds	[28]
285		(9*S*,6*Z*,10*E*,12*Z*)-9- hydroxy-6,10,12-octadecatrienoic acid	17871-22-6	$C_{18}H_{30}O_3$	Seeds	[28]
286		(2*E*)-2-dodecenoic acid/(*E*)-dodec-2-enoic acid	32466-54-9	$C_{12}H_{22}O_2$	Seeds, fruits	[28, 38]
287		(2*E*)-2-tetradecenoic acid/(*E*)- tetradec-2-enoic acid	26444-03-1	$C_{14}H_{26}O_2$	Seeds, fruits	[28, 38]
288		(2*E*,7*Z*,10*Z*,13*Z*)-hexadeca-2,7,10,13-tetraenoic acid		$C_{16}H_{24}O_2$	Fruits	[38]
289		(2*E*,7*Z*)-tetradeca-2,7-dienoic acid		$C_{14}H_{24}O_2$	Fruits	[38]
290		(11*R*)-hydroxyhexadeca-(2*E*,7*Z*,9*E*)-trienoic acid		$C_{16}H_{26}O_3$	Seeds	[28]
291		Pyrrole-2-carboxylic acid	634-97-9	$C_5H_5NO_2$	Fruits	[72]
292		2-methoxy-1, 4-biphenol-1-O- [6-O- (3-methoxy-4-hydroxybenzoyl)]-*β*-D-glucopyranoside		$C_{21}H_{24}O_{11}$	Fruits	[125]
